# SPOCK2 controls the proliferation and function of immature pancreatic β-cells through MMP2

**DOI:** 10.1038/s12276-024-01380-2

**Published:** 2025-01-01

**Authors:** Katarzyna Blaszczyk, Anna P. Jedrzejak, Natalia Ziojla, Ekaterina Shcheglova, Karolina Szarafin, Artur Jankowski, Christine A. Beamish, Jolanta Chmielowiec, Omaima M. Sabek, Ashok Balasubramanyam, Sanjeet Patel, Malgorzata Borowiak

**Affiliations:** 1 Institute of Molecular Biology and Biotechnology, Faculty of Biology, Adam Mickiewicz University, Uniwersytetu Poznanskiego 6, Poznan, 61-614 Poland; 2 Department of Surgery, Methodist Research Institute, Houston, TX 77030 USA; 3 Collegium Medicum, University of Warmia and Mazury, Aleja Warszawska 30, Olsztyn, 11-082 Poland; 4 Division of Diabetes, Endocrinology and Metabolism, Baylor College of Medicine, One Baylor Plaza, Houston, TX 77030 USA; 5 Keck School of Medicine, University of Southern California, 1975 Zonal Avenue, Los Angeles, CA 90033 USA

**Keywords:** Stem-cell differentiation, Cell proliferation

## Abstract

Human pluripotent stem cell-derived β-cells (SC-β-cells) represent an alternative cell source for transplantation in diabetic patients. Although mitogens could in theory be used to expand β-cells, adult β-cells very rarely replicate. In contrast, newly formed β-cells, including SC-β-cells, display higher proliferative capacity and distinct transcriptional and functional profiles. Through bidirectional expression modulation and single-cell RNA-seq, we identified SPOCK2, an ECM protein, as an inhibitor of immature β-cell proliferation. Human β-cells lacking SPOCK2 presented elevated MMP2 expression and activity, leading to β-integrin-FAK-c-JUN pathway activation. Treatment with the MMP2 protein resulted in pronounced short- and long-term SC-β-cell expansion, significantly increasing glucose-stimulated insulin secretion in vitro and in vivo. These findings suggest that SPOCK2 mediates fetal β-cell proliferation and maturation. In summary, we identified a molecular mechanism that specifically regulates SC-β-cell proliferation and function, highlighting a unique signaling milieu of SC-β-cells with promise for the robust derivation of fully functional cells for transplantation.

## SPOCK2 inhibition boosts stem cell derived beta cell growth

The human body has a limited ability to regenerate insulin-producing cells, which are crucial for managing diabetes. Researchers are exploring ways to create these cells from stem cells in the lab. This study focuses on understanding how to make these lab-grown cells more effective and plentiful. The researchers used a type of cell called SC-β-cells, derived from human stem cells to study how they grow and function. They discovered that a protein called SPOCK2 plays a role in controlling the growth of these cells. By reducing SPOCK2 levels, they found that the cells grew better and produced insulin more effectively. The study suggests that manipulating SPOCK2 and using proteins like MMP2 can help expand the number of functional insulin-producing cells. This summary was initially drafted using artificial intelligence, then revised and fact-checked by the author.

## Introduction

Insulin-producing pancreatic β-cells have a very limited capacity to regenerate in humans. Cadaveric islet transplantation has been offered as an experimental procedure to diabetic patients with labile glycemia or life-threatening hypoglycemia. However, the scarcity of donor tissues has hindered the widespread adoption of this treatment modality. The results from animal studies^1–3^ and preliminary findings from human clinical trials (NCT03163511^4,5^ and NCT04786262) suggest that physiological insulin replacement therapy for diabetes may be achievable by transplanting β like cells derived from human pluripotent stem cells (hPSCs).

Functional β-cells can be obtained by hPSC-directed differentiation in vitro^2^*;* therefore, hPSCs could theoretically provide a limitless supply of β-cells. However, despite significant progress in the development of pancreatic differentiation protocols, the derivation of therapeutically relevant, fully functional β-cells remain a complex and labor-, time- and cost-intensive process. Moreover, the end stage of pancreatic differentiation consists of a heterogeneous population of (1) immature and maturing β-cells, (2) other endocrine cell types and (3) nonendocrine cells, e.g., enterochromaffin cells^6–8^. Thus, cell therapy might ideally benefit from the use of an expandable population of human differentiated β-cells that maintain or even improve their functional capacity^9^. To this end, we need to better understand the mechanisms regulating immature human β-cell proliferation and maturation.

Genetic manipulation of cell cycle proteins can induce β-cell proliferation^10–13^, suggesting that the cell cycle is not irreversibly inactivated in human β-cells. Current viral delivery methods render this strategy unsuitable for therapeutic applications, as do issues related to target tissue specificity and lack of control over proliferation. The vast majority of mitogens that stimulate rodent β-cell proliferation are ineffective in humans^14–16^. The mitogens shown to be effective in humans are dual-specificity tyrosine phosphorylation-regulated kinase 1A (DYRK1A) inhibitors, including harmine^17–21^; inhibitors of both DYRK1A and glycogen synthase kinase 3β (GSK3β), such as GN7156 and GN4877^22^; and leukemia inhibitory factor (LIF)^23^. Furthermore, synergistic effects have been achieved by combining two or three mitogens (reviewed in ref. ^24^).

Human β-cell replication rates change throughout the cell lifetime, with a peak of 2% occurring during the first year after birth^25,26^. In adulthood, only 0.1–0.5% of β-cells replicate^26,27^, with the exception of specific conditions, such as pregnancy^28^. Given the increased proliferative capacity, here, we focused on immature (fetal and newborn) β-cells. Furthermore, SC-β-cells often resemble fetal β-cells more than adult β-cells, e.g., in their expression profile and lack well-regulated glucose-dependent insulin secretion^29–31^. Therefore, we hypothesized that mitogens that stimulate immature β-cell proliferation could also promote the expansion of SC-β-cells.

An analysis of our and other previously published single-cell RNA-sequencing (scRNA-seq) data from mouse pancreata at embryonic day (e) e14.5 and e16.5, when most endocrine progenitors (EPs) are formed and start to differentiate into β-cells and other endocrine cells^32,33^, identified the gene encoding an extracellular matrix (ECM) protein, SPARC (osteonectin), Cwcv and Kazal-like domains proteoglycan 2 (SPOCK2), as one of the genes expressed in newborn immature β-cells. SPOCK2 expression was low in early EPs and other pancreatic cell types but high in late-stage EP subpopulations and in newly formed Ins+ β-cells. Here, we demonstrate that SPOCK2 regulates the proliferation of human fetal β-cells, represented by SC-β-cells and the EndoC-βH1 line. Importantly, we showed that SPOCK2 deficiency also increases glucose-stimulated insulin secretion (GSIS) from β-cells. Matrix metalloproteinase-2 (MMP2) mimics the proliferative effect of genetic SPOCK2 inactivation in SC-β and EndoC-βH1 cells through activation of the β-integrin-JUN axis. Finally, we show that the long-term expansion of SC-β-cells by MMP2 treatment produces cells that are functional in vitro and in vivo with physiological competence comparable to that of human cadaveric islets. Together, these studies indicate a robust and previously unreported molecular mechanism that specifically regulates newly formed, immature β-cell replication and function that can generate the large and potent SC-β-cell pool needed for future regenerative strategies in diabetes, as well as for basic research and drug screening studies.

## Materials and methods

### EndoC-βH1 cell culture

The EndoC-βH1 cell line generated from the human fetal pancreas by Dr. Philippe Ravassard^34^ was obtained from the distributor Univercell-Biosolutions. The cells were cultured in Dulbecco’s modified Eagle’s medium containing 1 g/L glucose (DMEM; Corning) supplemented with 2% BSA fraction V fatty acid-free (Millipore), 50 μM 2-mercaptoethanol (Thermo Fisher Scientific), 10 mM nicotinamide (Sigma Aldrich), 5.5 μg/mL transferrin (Sigma Aldrich), 6.7 ng/mL selenite (Sigma Aldrich), 100 μ/mL penicillin, and 100 μg/mL streptomycin on Geltrex (200 μg/mL)- and fibronectin (4 μg/mL; Sigma Aldrich)-coated cell culture plates. The cells were tested for mycoplasma infection every two weeks. The plates were seeded at a density of 8 × 10^5^ cells/cm², and the cells were passaged every 7 days with trypsin (GIBCO). The medium was changed every 2–3 days. The cells were cultured at 37 °C in an atmosphere containing 5% CO_2_.

### hPSC culture and differentiation into β-cells

H1, HUES8 or HUES3 hPSCs were cultured in StemFlex (Thermo Fisher Scientific) medium on vitronectin (rh VTN; Thermo Fisher Scientific)-coated plates at 37 °C in an atmosphere containing 5% CO_2_. The cells were passaged every 3‒4 days (~80% confluency) with PBS-EDTA. The cells were tested for mycoplasma monthly via a PCR assay. Prior to differentiation, the cells were dispersed into single-cell suspensions with TrypLE Select (Thermo Fisher Scientific) and plated at a density of 1.5 × 10^5^ cells/cm^2^ in StemFlex supplemented with 10 µM Y-27632 (Peprotech) on Geltrex (Thermo Fisher Scientific)-coated plates. On subsequent days, the cells were washed in PBS, and the medium was changed as follows^35^.

Day 1: RPMI (Corning, USA) + 1× GlutaMAX (Thermo Fisher Scientific) + 1× pen/strep (Thermo Fisher Scientific) + 3 µM CHIR99021 (Peprotech) + 100 ng/mL activin A (Peprotech)

Days 2–3: RPMI + 1× GlutaMAX + 1× pen/strep+ 0.2% FBS HyClone (GE Healthcare) + Activin A

Days 4–5: RPMI + 2% FBS + 1 × GlutaMAX + 1 × pen/strep + 50 ng/mL KGF (Peprotech)

Days 6–9: DMEM (Corning) + GlutaMAX + 1× pen/strep + 1% B27 (Thermo Fisher Scientific) + 50 ng/mL KGF + 2 µM all-trans retinoic acid (Peprotech) + 100 nM LDN193189 (Peprotech) + 250 nM SANT-1 (Biotechne)

Days 10–14: DMEM (Corning) + 1× GlutaMAX + 1× pen/strep + 1% B27 (Thermo Fisher Scientific) + 100 nM LDN193189 + 250 nM SANT-1 + 1 µM phorbol 12,13-dibutyrate (Biotechne) + 1 µM Alk5i II (Adooq Bioscience)

Days 15–31: CMRL 1066 (Corning) + 1× GlutaMAX + 1 × pen/strep + 10% FBS + 10 µM Alk5i II + 1 µM T3 hormone (Sigma Aldrich).

The medium was changed daily on Days 1–19 and every other day from Day 20 onward.

### Treatment of β-cells with growth factors or small molecules

EndoC-βH1 or SC-β-cells were seeded on plates at a density of 8 × 10^5^ cells/cm² and cultured for 2‒3 days before treatment for 2‒7 days with 10 µM CHIR99021 (Peprotech), an MMP2 inhibitor (MMP2i) (Cayman Chemicals) at concentrations of 0.5 µM, 2 µM, and 5 µM; rh SPOCK2 (R&D Systems) at concentrations of 0.5 µg/mL, 1 µg/mL, and 2 µg/mL; rh MMP2 (R&D Systems) at concentrations of 5 ng/mL, 15 ng/mL, 30 ng/mL and 140 ng/mL; harmine (R&D Systems) at a concentration of 10 μM; GW788388 (R&D Systems) at a concentration of 2 μM; WS6 (R&D Systems) at a concentration of 1 μM; or the JNK inhibitor SP600125 (Tocris) at concentrations of 20 μM or 40 μM.

### Human islet isolation

Human pancreata were obtained with informed consent for transplant or research use from relatives of heart-beating, cadaveric multiorgan donors through the efforts of The National Disease Research Interchange (NDRI), Tennessee Donor Services, the Mid-South Transplant Foundation, and the United Network for Organ Sharing. Human islets were isolated under Human Islet Isolation for Research (HIIR) ethical approval number Pro00001097 to Drs. Omaima Sabek and Daniel Fraga (Houston Methodist Research Institute). Donor demographics were collected at the time of acceptance and included age (in years), sex, race, body mass index, history of alcohol intake, and history of hypertension. Donor-related laboratory data, including donor blood glucose, serum amylase, lipase, liver function tests (ALT, AST), cytomegalovirus infection status, and procurement and preservation parameters, such as pancreatic warm and cold ischemia times, ventilation time, pancreas weight and adequacy of pancreas perfusion, were also recorded. The pancreas was perfused with University of Wisconsin (UW) solution. Human islets were isolated from cadaver donors via an adaptation of the automated method described by Ricordi et al.^36^. Liberase was dissolved in cold (4 °C) Hank’s balanced salt solution (HBSS) (Mediatech, Inc., Herndon, VA) supplemented with 0.2 mg/ml DNase (Sigma Chemical Co., St. Louis, MO), 1% penicillin‒streptomycin (Sigma Chemical Co.), 20 mg/dl calcium chloride (J.T. Baker, Inc., Phillipsburg, NJ), and HEPES (Sigma Chemical Co.). After dissolution, the pH was adjusted to between 7.7 and 7.9. The enzyme preparation was then sterile filtered, warmed to 37 °C, and used for intraductal distension of the pancreas. The distended pancreas was cut into several pieces and placed in a Ricordi chamber, and the heating circuit was started. Pancreatic digestion was performed at 37 °C until more than 90% free islets were observed in the sample. The digested tissue was collected in cold HBSS supplemented with 20% human serum and 1% penicillin‒streptomycin solution and centrifuged at 400 × *g* at 4 °C for 5 min. The tissue pellets were pooled into cold UW solution and held at 4 °C for 1 h with periodic mixing. Islet purification was performed on a COBE 2991 Cell Processor (COBE BCT, Lakewood, CO) using OptiPrep (Nycomed Pharma AS, Oslo, Norway) as a step gradient based on a modification of the procedure of Robertson et al.^37^. Islet culture: Aliquots from human islet isolations were cultured in SFM containing 1% ITS (Collaborative Biomedical Products, Bedford, MA), 1% L-glutamine (Life Technologies, Gaithersburg, MD), 1% antibiotic-antimycotic solution (Sigma Chemical Co.), and 16.8 µM zinc sulfate, as described previously^38^. Human islets were isolated from 5 nondiabetic donors: 49-year-old (BMI 25.3, A1c 4.9%), 52-year-old (BMI 37.5, A1c 5.3%), 63-year-old (BMI 32.5, A1c 4.8%) and 69-year-old (BMI 33.3, A1c 5.5%) males and 68-year-old (BMI 28.4, A1c 4.6%) and 70-year-old (BMI 26.1, A1c 4.8%) females.

### Mouse islet isolation

Mouse islet isolation was performed via common bile duct perfusion with ice-cold 0.8 mM collagenase P (Roche), dissection, additional gentle dissociation with collagenase P and further purification in Histopaque gradient (Sigma) as described previously^39^. For each islet prep, 10 adult (6–12-month-old) ICR males and females were used.

### Glucose-stimulated Insulin secretion

Human primary, islet, SC-β-cells or EndoC-βH1 cells were incubated for 1 h in Krebs buffer (128 mM NaCl, 5 mM KCl, 2.7 mM CaCl_2_, 1.2 mM MgCl_2_, 1 mM Na_2_HPO_4_, 1.2 mM KH_2_PO_4_, 5 mM NaHCO_3_, 10 mM HEPES, 0.1% BSA) and then incubated for 30 min in low-glucose (2.8 mM D-(+)-glucose) or high-glucose (16.7 mM D-(+)-glucose) Krebs buffer for each step. After each incubation step, the supernatants were collected. The cells were then dissociated via TrypLE Express and quantified (Countess, Invitrogen), and human insulin was measured via ELISA (Mercodia) according to the manufacturer’s instructions.

### Reverse transcription‒quantitative PCR (RT‒qPCR)

Total RNA was extracted via TRIzol reagent (Thermo Fisher Scientific) according to the manufacturer’s instructions. For cDNA synthesis, 500 ng of RNA was reverse transcribed with the SuperScript II Reverse Transcriptase cDNA Synthesis Kit (Thermo Fisher Scientific) along with random hexamer primers. The cDNA was PCR amplified with Power SYBR Green qPCR Master Mix (Thermo Fisher Scientific). To quantify the gene expression levels, the amount of target gene in each sample was normalized against that of β-ACT via the ΔCT quantification method^40^. The primer sequences are listed in Table 1.

**Table 1:** RT‒qPCR primers.

Primer name	Sequence
SPOCK2_F	CTGGCCGAAGGCGACGCCAA
SPOCK2_R	CGTCTCGGAAGCGGTTCCAG
β-ACT_F	ACAGAGCCTCGCCTTTGCCGAT
β-ACT_R	ATCATCCATGGTGAGCTGGCGG
TIMP1_F	TCTGGCATCCTGTTGTTGCT
TIMP1_R	CGCTGGTATAAGGTGGTCTGG
MMP2_F	ACATCAAGGGCATTCAGGAG
MMP2_R	TGAACCGGTCCTTGAAGAAG
GADPH_F	TCAAGGCTGAGAACGGGAAG
GADPH_R	CGCCCCACTTGATTTTGGAG
INS_F	ACGAGGCTTCTTCTACACACCC
INS_R	TCCACAATGCCACGCTTCTGCA
CHGA_F	GGTTCTTGAGAACCAGAGCAGC
CHGA_R	GCTTCACCACTTTTCTCTGCCTC
NKX6-1_F	CCTATTCGTTGGGGATGACAGAG
NKX6-1_R	TCTGTCTCCGAGTCCTGCTTCT
SUR1_F	GACGACAAGAGGACAGTGGTCT
SUR1_R	GCATTCAGACCTCTGGAAGTCC
KIR6-2_F	TGTGTCACCAGCATCCACTCCT
KIR6-2_R	GTTCTGCACGATGAGGATCAGG

### Immunofluorescence staining

Human pancreas sections were processed at Baylor College of Medicine, Houston, TX (USA), under the IRB-3097 approval granted to Malgorzata Borowiak. The donor identities were encrypted, and the data were analyzed anonymously. Human 10.6- and 13-week fetal pancreas samples were fixed in 4% paraformaldehyde/phosphate-buffered saline (PBS) for 4 h, washed with PBS, soaked in 30% sucrose, and embedded in TissueTek. Section (12 μm thick) were cut onto Superfrost Plus-coated glass slides and stored at –80 °C.

EndoC-βH1 cells, SC-β-cells, and human or mouse primary islets were fixed by incubation with 4% paraformaldehyde/PBS for 15 min at room temperature and subjected to three short washes in PBS. Fixed cells and tissue samples were permeabilized via incubation with 0.5% Triton X-100 (BioShop) in PBS for 15 min and blocked via incubation with 3% BSA (Sigma Aldrich) or 0.1% Tween-20 (BioShop) in PBS for 30 min at room temperature or with 3% normal donkey serum (Jackson ImmunoResearch) or 0.1% Triton X-100 in PBS (NDS). The samples were then incubated overnight at 4 °C with primary antibodies diluted in 5% NDS. The primary antibodies used in the study are listed in Table 2. After two 10-min washes with 0.1% Tween 20 in PBS, the cells were incubated with secondary antibodies conjugated with Alexa Fluor 488, TRITC or Alexa Fluor 647 (all from Jackson ImmunoResearch) diluted 1:800 in 5% NDS for 2 h at room temperature. The excess secondary antibody was removed by two washes for 5 min each with PBS-Tween, and the samples were then incubated with DAPI (Sigma Aldrich) as a counterstain. The tissue samples were mounted in ProLong Diamond Antifade (Thermo Fisher Scientific) before imaging.

**Table 2:** Antibodies used for immunofluorescence staining.

Antibody name	Company	Catalog	Dilution	Primary/Secondary	Host	Reactivity
CHGA	Santa Cruz, CA	sc-393941	1:200	Primary	Mouse	Hu, Mus, Rat
C-peptide	DSHB	GN-ID4	1:40	Primary	Rat	Hu
GCG	Santa Cruz, CA	sc-514592	1:100	Primary	Mouse	Hu, Mus, Rat
Ki67	BD Pharmingen	556027	1:100	Primary	Mouse	Hu, Mus, Rat
PDX1	R&D	AF2419	1:100	Primary	Goat	Hu
pHH3	Millipore	06-570	1:100	Primary	Rabbit	Hu, Mus
SPOCK2	Sigma Aldrich	HPA044605	1:50	Primary	Rabbit	Hu
c-JUN	Cell Signaling	9165	1:300	Primary	Rabbit	Hu, Mus, Rat, Mk
phospho-c-JUN (S73)	Cell Signaling	3270	1:600	Primary	Rabbit	Hu, Mus, Rat, Mk, Pg
PCSK1	Abcam	Ab220363	1:100	Primary	Rabbit	Hu, Mus, Rat
CD49a- Alexa 488 conjugated	R&D	FAB56761G	1:100	Primary	Rabbit	Hu
				+Secondary		
Ki67-Alexa488 conjugated	R&D	IC7617G	1:100	Primary	Rabbit	Hu
				+Secondary		
TM4SF- APC conjugated	R&D	FAB7998A	1:50	Primary	Mouse	Hu
				+Secondary		
Alexa Fluor 488	Jackson ImmunoResearch	715-545-150	1:800	Secondary	Donkey	anti-Mouse IgG (H + L)
Alexa Fluor 488	Jackson ImmunoResearch	711-545-152	1:800	Secondary	Donkey	anti-Rabbit IgG (H + L)
Alexa Fluor 488	Jackson ImmunoResearch	705-545-147	1:800	Secondary	Donkey	anti-Goat IgG (H + L)
TRITC	Jackson ImmunoResearch	705-025-147	1:800	Secondary	Donkey	anti-Goat IgG (H + L)
TRITC	Jackson ImmunoResearch	715-025-150	1:800	Secondary	Donkey	anti-Mouse IgG (H + L)
TRITC	Jackson ImmunoResearch	711-025-152	1:800	Secondary	Donkey	anti-Rabbit IgG (H + L)
TRITC	Jackson ImmunoResearch	712-025-153	1:800	Secondary	Donkey	anti-Rat IgG (H + L)
Alexa Fluor 647	Jackson ImmunoResearch	705-605-147	1:800	Secondary	Donkey	anti-Goat IgG (H + L)
Alexa Fluor 647	Jackson ImmunoResearch	711-605-152	1:800	Secondary	Donkey	anti-Rabbit IgG (H + L)
Alexa Fluor 647	Jackson ImmunoResearch	715-605-151	1:800	Secondary	Donkey	anti-Mouse IgG (H + L)

*Hu* human, *Mus* mouse, *Mk* monkey, *Pg* Pig.

### Imaging

Images were obtained with an epifluorescence or confocal microscope. Epifluorescence microscopy was performed with a Leica DM IL-Led (Leica, Germany) microscope with N Plan Fluor 4x/0.12, N Plan Fluor 10x/0.30, N Plan Fluor 20x/0.40 and N Plan Fluor 40x/0.60 lenses and a JENOPTIK Progres Gryphax camera (JENOPTIK, Germany). Confocal microscopy was performed with a Nikon A1Rsi (Nikon, Germany) microscope with Plan Fluor 4x/0.13, Plan Apo 10x/0.45 DIC N1, Plan Apo VC 20x/0.75 DIC N2, Apo 40x/1.25 WI λS DIC N2, and Plan Apo VC 60x/1.4 Oil DIC N2 lenses and with Nikon NIS Elements AR 5.21.01 64-bit software (Nikon, Germany). Mean gray value normalization was achieved by dividing the mean fluorescence signal of the protein target of interest by the mean fluorescence signal of DAPI.

### Western blot

Protein extracts were prepared from EndoC-βH1 cells in radioimmunoprecipitation assay (RIPA) buffer (50 mM Tris-HCl, pH = 8.0 (Bio-Shop), 150 mM NaCl (Bio-Shop), 1% Nonidet-40 (Bio-Shop), 0.5% sodium deoxycholate (Bio-Shop), 0.1% SDS (Bio-Shop), 1% protease inhibitor cocktail (Sigma Aldrich), 1% EDTA (Sigma Aldrich), and 0.1% PMSF (Sigma Aldrich)) and stored at −80 °C. Protein concentrations were determined with a bicinchoninic acid (BCA) kit (Pierce). Thirty micrograms of protein in Bolt LDS buffer (Thermo Fisher Scientific) were heated at 70 °C for 10 min and then loaded onto a 4–12% Bis-Tris Plus gel (Thermo Fisher Scientific). Electrophoresis was performed at 165 V for the first 10 min and then at 200 V for 30 min, with a constant current of 100 amps. The resulting bands were transferred onto PVDF (Thermo Fisher Scientific) membranes for 10 min, and the membranes were then blocked with 0.125% nonfat dry milk or 1% BSA in TBS-Tween 20 and incubated with primary antibodies against SPOCK2, c-JUN, phospho-c-JUN, α-TUBULIN or GAPDH (Table 3). HRP-conjugated secondary antibodies—anti-rabbit (Sigma Aldrich, A9169) 1:20,000 or anti-mouse (Sigma Aldrich, A9044) 1:20,000—were used as appropriate. Antibody‒antigen complexes were visualized by enhanced chemiluminescence (ECL) with the Luminata Forte HRP Substrate (Merck Millipore) and detected with the G:Box System (Syngene). The western blot data were quantified via ImageJ with the “ analyze gel” function.

**Table 3:** Antibodies used for western blotting.

Antibody name	Company	Catalog	Dilution	Diluted in	Incubation time
SPOCK2	Sigma Aldrich	HPA044605	1:500	0.125% nonfat dry milk	O/N
c-JUN	Cell Signaling	9165	1:1000	0.125% nonfat dry milk	O/N
phospho-c-JUN (S73)	Cell Signaling	3270	1:1000	1% BSA in TBS-T	O/N
phospho-FAK (T397)	Cell Signaling	8556	1:300	0.125% nonfat dry milk	O/N
α-tubulin	Merck Millipore	04-1117	1:2000	0.125% nonfat dry milk	20 min
GAPDH	Merck Millipore	MAB374	1:2000	0.125% nonfat dry milk	20 min
anti-rabbit	Sigma Aldrich	A9169	1:20000	0.125% nonfat dry milk	20 min
anti-mouse	Sigma Aldrich	A9044	1:20000	0.125% nonfat dry milk	20 min

*O/N* overnight.

### Zymography

For gelatin zymography, 10% SDS‒PAGE gels were impregnated with 4 mg/ml soluble gelatin type A substrate. The cell supernatants were collected and concentrated after 24 h of culture, prepared with a nonreducing loading dye (0.01% bromophenol blue, 10% SDS, 125 mM Tris-HCl, 20% glycerol) and separated via electrophoresis at 150 V for 1 h at 4 °C. The gels were washed in renaturing buffer and incubated in assay buffer overnight. Renaturing buffer contained 50 mM Tris-HCl buffer at pH 7.4 and 2.5% Triton X-100 with 5 mM CaCl_2_ and 1 μM ZnCl_2_. The assay buffer contained 50 mM Tris-HCl and 1% Triton X-100 with 5 mM CaCl_2_ and 1 μM ZnCl_2_. The gels were stained for 2 h in 0.5% Coomassie blue stain with 10% acetic acid and 40% methanol, destained with 10% acetic acid and 40% methanol, and imaged. Densitometric quantification of white band intensity (indicating protease activity) was performed via ImageJ.

### Cell counting

For the proliferation assay, cell quantification was performed with ImageJ software (https://imagej.nih.gov/ij/). The bioimages were subjected to binarization, and an optimal signal threshold and watershed function were applied such that the cells in the image were distinguishable objects. The cells were then counted with the “Analyze particles” function for the blue (corresponding to DAPI^+^ cells) and red (corresponding to pHH3^+^ or Ki67^+^ cells) channels separately. For validation of the results, quantification was performed via the Manders colocalization method^41^ with the ImageJ “Just Another Colocalization” plugin (JACoP)^42^ (https://imagej.nih.gov/ij/plugins/track/jacop.html).

### RNA sequencing

We used 1000 ng of total RNA isolated from EndoC-βH1 WT, SPOCK2 KD, SPOCK2 OE and GIPZ cells to prepare libraries with the TruSeq RNA Library Prep Kit v2 according to the manufacturer’s protocol. Libraries were prepared in duplicate. Libraries were quantified with a Qubit fluorometer (TFS), and their quality was assessed with an Agilent High Sensitivity DNA Kit (Agilent Technologies). Libraries were sequenced with an Illumina HiScanSQ sequencer. RNA-Seq raw paired-end reads were trimmed with fastp^43^. The trimmed reads were aligned with the Ensembl GRCh38 human genome with STAR (v2.7)^44^^,^ and counts were obtained with featureCounts v1.6.3^45^. The raw RNA-seq sequence reads were analyzed via the iDEP9.4 (integrated differential expression and pathway analysis) interactive platform (http://bioinformatics.sdstate.edu/idep93/)^46^. Genes differentially expressed between EndoC-βH1 SPOCK2-KD and GIPZ cells or between SPOCK2-OE and WT cells were identified and normalized with the DESeq2 package^47^. The RNA-seq data were deposited in the NCBI GEO database under accession number GSE190361. Differentially expressed genes (DEGs) significantly (*p*-value ≤ 0.05) upregulated with a log2FC ≥ 0.5 or downregulated with a log2FC ≤ –0.5 in any of the sequenced samples were selected for further analysis. Cluster analysis was performed with Genesis software (http://genome.tugraz.at/genesisclient/genesisclient_description.shtml)^48^. The average linkage method was used for hierarchical clustering. The induction ratios of genes were log_2_-transformed and subjected to clustering analysis. The automatic gene cluster assignment method was used to create gene clusters. Volcano plots were plotted with GraphPad Prism 8 software for genes with normalized reads, with –log_10_ adjp values on the *y*-axis and log_2_FC values on the *x*-axis. Genes with –log10 adjp ≥ 2 and log2FC ≥ 0.5 or log2FC ≤ –0.5 were considered to be significantly differentially expressed. Enrichment in Gene Ontology (GO) categories was assessed with Gorilla software (http://cbl-gorilla.cs.technion.ac.il/)^49^. A *p*-value of 10^–3^ was used as a threshold, and Illumina gene lists from HumanHT-12 v4 were used as the background model. All statistically significant and enriched GO categories were analyzed with Revigo software (http://revigo.irb.hr/)^50^. Only GO terms with *p* values ≤ 0.05 were considered significantly enriched. Kyoto Encyclopedia of Genes and Genomes (KEGG) and Wiki pathway analyses were performed to obtain systematic and comprehensive information further identifying potential pathways among the DEGs. The GeneCodis interactive platform (https://genecodis.genyo.es/)^51^ was used for functional enrichment analysis. The cutoff value for significant KEGG and Wiki pathway results was an adjusted *p*-value below 0.05. To identify overrepresented transcription factor-binding site motifs in sequences from coregulated or coexpressed genes, Pscan^52^ was used with the selected promoter region –950 to +50 bp from the TSS and the TRANSFAC matrix sequence. Gene set enrichment analysis (GSEA) was performed with GSEA v4.1.0 software (http://software.broadinstitute.org/gsea/msigdb/index.jsp)^53^. GSEA identifies functional enrichment by comparing genes with predefined gene sets. A gene set is a set of genes with similar localizations, pathways, functions, or other features. The input data for the GSEA were normalized counts for the SPOCK2 KD vs. GIPZ or SPOCK2 OE vs. WT gene sets; a mapping file for identifying probe sets; and a reference gene set based on the Molecular Signatures Database set: h.all.v7.4.symbols.gmt (Hallmarks) or a custom-built gene set of β-integrin signaling pathway-related genes used for enrichment analysis. The permutation number was set to 1000. The enrichment gene sets with a *p* value ≤ 0.05 and a false discovery rate (FDR) ≤ 0.25 in the GSEA were considered to exhibit statistically significant differences. We used the default parameters of GSEA software. An enrichment map was used to visualize the GSEA results. The enrichment score (ES) and FDR values were used to sort hallmark and β-integrin pathway-enriched gene sets. The set of genes significantly upregulated in SPOCK2-KD cells was compared via Venn diagram analysis (http://bioinfogp.cnb.csic.es/tools/venny/index.html)^54^ with the set of genes downregulated in SPOCK2-OE cells.

### Single-cell RNA sequencing

The SC-WT and SPOCK2 KO β-cell spheroids were dispersed, washed for flow cytometry, and filtered through a 30 μm mesh filter. Afterward, the cells were counted via Countess and adjusted to 700–1200 cells/μl. scRNA-seq was performed with the 10× Genomics platform and Illumina according to the manufacturer’s protocol. Libraries were pair-end sequenced with a depth of 40,000 reads per cell. The raw scRNA-seq data were processed with bcl2fastq v2.20 to demultiplex samples and convert the .bcl files to .fastq format. For initial data processing and some basic analyses, 10x Genomics Cell Ranger pipelines and the Seurat R package^55^ were used. Cell Ranger incorporates the STAR RNA-seq aligner, reads were aligned to the hg38 reference genome and filtered, unique molecular identifiers were counted, and gene‒barcode matrices were generated for each cell. WT and KO data aggregation was performed with the “cellranger agrr” algorithm. To identify distinct cell populations on the basis of shared and unique patterns of gene expression, we performed κ-means and graph-based clustering. Prior to clustering, principal component analysis (PCA) was run to determine the meaningful variance before both. Single-cell data visualization with the UMAP algorithm, analysis of DEGs, violin plots and bubble plots with gene expression patterns were performed with either Loupe Browser 6.2.0 software (10x Genomics, USA) or R Studio with the Seurat package. To model the relationship between gene expression and the S and G2M cell cycle scores on the basis of canonical markers, the Cell-Cycle Scoring algorithm provided in Seurat package was used^56^. In short, the algorithm assigns each cell a score on the basis of the expression of genes associated with the S and G2M phases of the cell cycle. For the threshold, cells with S and G2M scores exceeding the median values for the SC-β-cell cluster were classified as proliferating. Cells with both S scores and G2M scores below the median scores for their respective phases were classified as G1 phase.

### Lentivirus production

HEK293T cells were used to seed 10 cm^2^ plates one day before transfection at 70% confluence in DMEM (Corning) supplemented with 10% FBS (GE Healthcare) and 1× penicillin/streptomycin medium. The pGIPZ-based shRNA plasmids for SPOCK2 knockdown (SPOCK2 KD) were obtained from the Cell-Based Assay Screening Core at the Baylor College of Medicine, Houston, TX, USA. We assessed the efficiency of two different SPOCK2 shRNAs. The following hairpin sequence was used to establish the stable SPOCK2-KD cell line:

TGCTGTTGACAGTGAGCGACGTGAAACTCCATGGAAACAATAGTGAAGCCACAGATGTATTGTTTCCATGGAGTTTCACGGTGCCTACTGCCTCGGA

For lentivirus production, the packaging vectors psPAX2 (12 μg) and pMDG (6 μg) and 18 μg of SPOCK2 shRNA plasmid (SPOCK2 KD) or the GIPZ control plasmid encoding a scrambled shRNA were used. HEK293T cells were transfected with plasmids via Lipofectamine 3000 (Thermo Fisher Scientific) in accordance with the manufacturer’s protocol. The transfected cells were incubated for 6 h, and the medium was then replaced with DMEM (Corning) supplemented with 10% FBS (GE Healthcare) and 1× penicillin/streptomycin. The medium was replaced on each of the next three days. The supernatants were collected and passed through a 0.45 μm filter (Millipore), and the viral particles were concentrated by ultracentrifugation at 98,250 × *g* for 2 h at 4 °C, with the centrifuge brakes off. The viral pellets were resuspended in 200 μL of sterile PBS and stored at –80 °C.

### Lentiviral transduction and cell isolation

Two days before lentiviral transduction, EndoC-βH1 cells were seeded in six-well plates at a density of 8 × 10^5^ cells/cm^2^. For transduction, the cell medium was supplemented with 8 μg/mL polybrene (Sigma‒Aldrich) and 50 μL of concentrated virus suspension: either SPOCK2 KD or GIPZ. The cells were incubated at 37 °C in an atmosphere containing 5% CO_2_ for 72 h. The medium was then replaced with fresh medium of the same composition supplemented with 3 μg/mL puromycin. Antibiotic selection was performed over a period of two weeks.

### SPOCK2 OE construct

SPOCK2 cDNA was amplified from total EndoC-βH1 cDNA with the following primers:

hSPOCK2_OE_FOR:AGCGCTACCGGACTCAGATCATGCGCGCCCCGGGCTGCGG hSPOCK2_OE_REV:CACGCGTCATGGTGGCGGCGGACCAGATGTAGCCCCCGTCGTCAGCCTC

The primers were designed with the Benchling Assembly Wizard tool (https://benchling.com). SPOCK2 cDNA was amplified via PCR with Q5 polymerase (NEB) according to the manufacturer’s instructions, with touchdown PCR cycling conditions^57^. The cycling conditions were as follows: 98 °C for 30 s, followed by 16 cycles of 98 °C for 20 s, 72 °C (−0.5°C/cycle) for 20 s, and 72 °C for 30 s; 30 cycles of 98 °C for 20 s, 70 °C for 20 s, and 72 °C for 30 s; and a final extension step at 72 °C for 5 min. The resulting SPOCK2 PCR product was isolated with the Monarch DNA Gel Extraction Kit (NEB). The pEGFP_N1_FLAG vector^58^ was a gift from Dr. Patrick Calsou (Addgene, #60360). This vector was digested with the *Bam*HI and *Bgl*II restriction enzymes (Thermo Fisher Scientific). Then, NEBuilder HiFi DNA Assembly Cloning (NEB) was used to ligate the SPOCK2 PCR product after verification by Sanger sequencing. EndoC-βH1 cells were transfected with the plasmid with Lipofectamine 3000 (Thermo Fisher Scientific) according to the manufacturer’s protocol.

### Transient transfection with siRNA

EndoC-βH1 cells were transfected with MISSION esiRNA targeting human SPOCK2 (Sigma‒Aldrich) using Lipofectamine 3000 (Thermo Fisher Scientific) according to the manufacturer’s protocol. We used 300 ng of esiRNA/well to transfect cells growing on a 48-well plate; 72 h post transfection, the EndoC-βH1 cells were fixed and stained.

The cDNA esiRNA target sequence was as follows:

CCGTGAAACTCCATGGAAACAAAGACTCCATCTGCAAGCCCTGCCACATGGCCCAGCTTGCCTCTGTCTGCGGCTCAGATGGCCACACTTACAGCTCTGTGTGTAAGCTGGAGCAACAGGCGTGCCTGAGCAGCAAGCAGCTGGCGGTGCGATGCGAGGGCCCCTGCCCCTGCCCCACGGAGCAGGCTGCCACCTCCACCGCCGATGGCAAACCAGAGACTTGCACCGGTCAGGACCTGGCTGACCTGGGAGATCGGCTGCGGGACTGGTTCCAGCTCCTTCATGAGAACTCCAAGCAGAATGGCTCAGCCAGCAGTGTAGCCGGCCCGGCCAGCGGGCTGGACAAGAGCCTGGGGGCCAGCTGCAAGGACTCCATTGGCTGGATGTTCTCCAAGCTGGACACCAGTGCTGACCTCTTCCTGGA

### Deletion of the SPOCK2 (KO) gene in hPSCs

sgRNAs targeting exon 1 of *SPOCK2* were designed via Benchling software. The sgRNA sequences are listed below:

sgRNA_SPOCK2_1 CTGGCCGAAGGCGACGCCAA

sgRNA_SPOCK2_2GTCCTCCATGAAATTGCCGG sgRNA_SPOCK2_3 CTTGCCGCTGTACTGCGAGA

The sgRNAs were produced in-house. The PCR template consisted of 120-nucleotide single-stranded DNA, including a T7 promoter, sgRNA target-specific sequences, and constant sgRNA sequences. The PCR product was used for in vitro transcription to generate sgRNAs. On the day of transfection, doxycycline-treated HUES8-iCas9 hPSCs were dispersed into single cells via TrypLE Express, counted, and seeded with medium supplemented with ROCKi into Geltrex-coated 24-well plates at a density of 1.5 × 10^5^ cells/well. The cells were reverse transfected via Lipofectamine RNAiMax (Thermo Fisher Scientific, Netherlands). The medium was changed the next day, and the cells were cultured for an additional 24 h. Subsequently, the cells were plated at single-cell density and cultured for 7 days. After 7 days, selected colonies grown from a single cell were picked. PCR of genomic DNA isolated from the clones with primers flanking the CRISPR-edited region and Sanger sequencing allowed the identification of clones with homozygous KO mutations in the *SPOCK2* gene. CRISPR editing efficiency was calculated via the bioinformatic tool Inference of CRISPR Edits (ICE; synthego.com, USA) on the basis of DNA-sequencing data comparisons of edited and control samples.

### IncuCyte analysis

An IncuCyte live cell imager (Sartorius) was used to track living cells. EndoC-βH1 SPOCK2 KD, SPOCK2 OE, GIPZ control and WT cells were seeded into 96-well plates, which were subsequently incubated for seven days at 37 °C in an atmosphere containing 5% CO_2_. Photomicrographs were taken every four hours, and the confluence of the cultures was measured in real time with IncuCyte Base Analysis Software (Sartorius). Cell proliferation was monitored by analyzing the area of the image occupied by cells (% confluence) over time. We also used the IncuCyte Cell-by-Cell Analysis Software Module to count the number of phase objects over time as an alternative method of quantifying cell proliferation.

### Electron microscopy

To analyze the granular ultrastructure, EndoC-βH1 was fixed at RT for 2 h in a mixture containing 1.25% PFA, 2.5% glutaraldehyde, and 0.03% picric acid in 0.1 M sodium cacodylate buffer (pH 7.4). The samples were then washed in 0.1 M cacodylate buffer and postfixed at RT with a mixture of 1% OsO_4_/1.5% KFeCN_6_ once for 2 h and then once for 1 h. After washing with water, the samples were stained in 1% aqueous uranyl acetate for 1 h, washed, and subsequently dehydrated. A 1 h incubation in propylene oxide was followed by infiltration overnight in a 1:1 mixture of propylene oxide and TAAB Epon, after which the samples were embedded in TAAB Epon. The cut sections were then stained with 0.2% lead citrate. A JEOL 1200EX transmission electron microscope or a TecnaiG2 Spirit BioTWIN was used to analyze the samples.

### In vivo transplantation of SC-derived β-cells and human islets

In brief, saline (sham-operated) or cells were injected into the kidney capsules of ~6-week-old (at least 21 g) male SCID-Beige animals (Harlan). The animals were anesthetized with Avertin (250 mg/kg) delivered intraperitoneally under aseptic conditions. The surgical site was shaved and disinfected with alcohol and betadine. Using a syringe, ~50 μl of the cell mixture was injected under the capsule of the left kidney. After surgery, the animals were administered 5 mg/kg carprofen for 2 days. The mice were housed individually and observed for the appearance of visible tumors. At 3 weeks post surgery, the mice were subjected to glucose tolerance tests (GTTs). All animal experiments were performed in accordance with the International Animal Care and Use Committee (IACUC) regulations under the animal protocol AN-6037.

### STZ-induced diabetes in mice

Some mice with SC-β-cells transplanted under the kidney capsule received one intraperitoneal injection of streptozotocin (150 mg/kg) and underwent GTTs two months later.

### Glucose tolerance test and glucose-stimulated human C-peptide secretion in vivo

The mice were fasted overnight (16 h) with water only in cages in which wire mesh flooring separated the animal from its bedding. Glucose was injected intraperitoneally (3 g/kg), and blood samples were taken from the tail and collected into heparin-coated tubes (BD) before (0) and 15, 30 and 60 min after glucose injection. A handheld glucometer (Accu-Chek, Roche) was used to measure blood glucose levels.

Before (0) and at 15, 30 and 60 min after glucose injection, blood samples were collected and spun, and the supernatant was stored at −80 °C. The levels of human C-peptide were measured via an ELISA specific to human, not mouse, C-peptide (ultrasensitive C-peptide human ELISA; Mercodia). Age-matched controls included samples from animals that had received saline only (sham-operated) or human islets (positive controls) and that had been subjected to GTTs in parallel. To verify the functionality of the SC-β-cell graft, the engrafted kidney was removed 4 months after streptozotocin (STZ) injection. The kidney was removed after the renal vein, artery and ureter were ligated. Following removal, the mice were fasted, and their glucose levels were measured. Mice with blood glucose >20 mmol/l were treated with daily insulin (0.5 μ/kg) after glucose measurement.

### FACS analysis

The cells were dissociated with trypsin to obtain a single-cell suspension and fixed by incubation with 4% paraformaldehyde in PBS supplemented with 0.1% saponin for 30 min at room temperature. The samples were washed once with 0.1% saponin–1% BSA–PBS (SBP). The resuspended cells were then incubated overnight at 4 °C on a roller with an anti-SPOCK2 (Sigma Aldrich) antibody diluted in SBP. The following day, the cells were washed twice with SBP and incubated with secondary antibodies conjugated with TRITC or Alexa Fluor 647 (Jackson ImmunoResearch) diluted 1:800 in SBP for 1 h at room temperature. The excess secondary antibodies were removed by washing once with PBS, and the cells were resuspended in PBS and used directly for FACS analysis. Flow cytometry data were acquired with a CytoFLEX Flow or Aria II flow cytometer (Beckman Coulter) or CYTEK Aurora. Flow cytometry data analysis, including gating, quantification, and the generation of density plots/histograms, was performed with CytoExpert v2.4 or Aria software (Beckman Coulter) or CYTEK Aurora. FACS enrichment for CD49a^+^ and TM4SF4^-^ was performed on SC-β-cells. The cells were plated on laminin-coated plates overnight for recovery.

### Statistics

All the graphs were prepared in GraphPad Prism 8. For statistical analyses, unpaired two-tailed Student’s *t* tests or one-way ANOVA for multiple comparisons were performed. All values are shown as the means ± SDs.

## Results

### SPOCK2 is expressed in human β-cells in vivo and in vitro

Our previous scRNA-seq of the mouse pancreas revealed temporal biases in endocrine cell differentiation, with e14.5 and e16.5 EPs preferentially forming α- and β-cells, respectively^33^. From this dataset, we queried for genes specifically expressed in developing β-cells and identified *Spock2*, which was expressed in early β-cells and only in a small subset of e14.5 late EPs (Supplementary Fig. 1a), whereas it was present in a substantial number of e16.5 late EPs (Supplementary Fig. 1b). This temporally biased expression pattern suggests that *Spock2* might regulate EP commitment to β-cell fate and the expansion or survival of fetal β-cells.

**Fig. 1:**
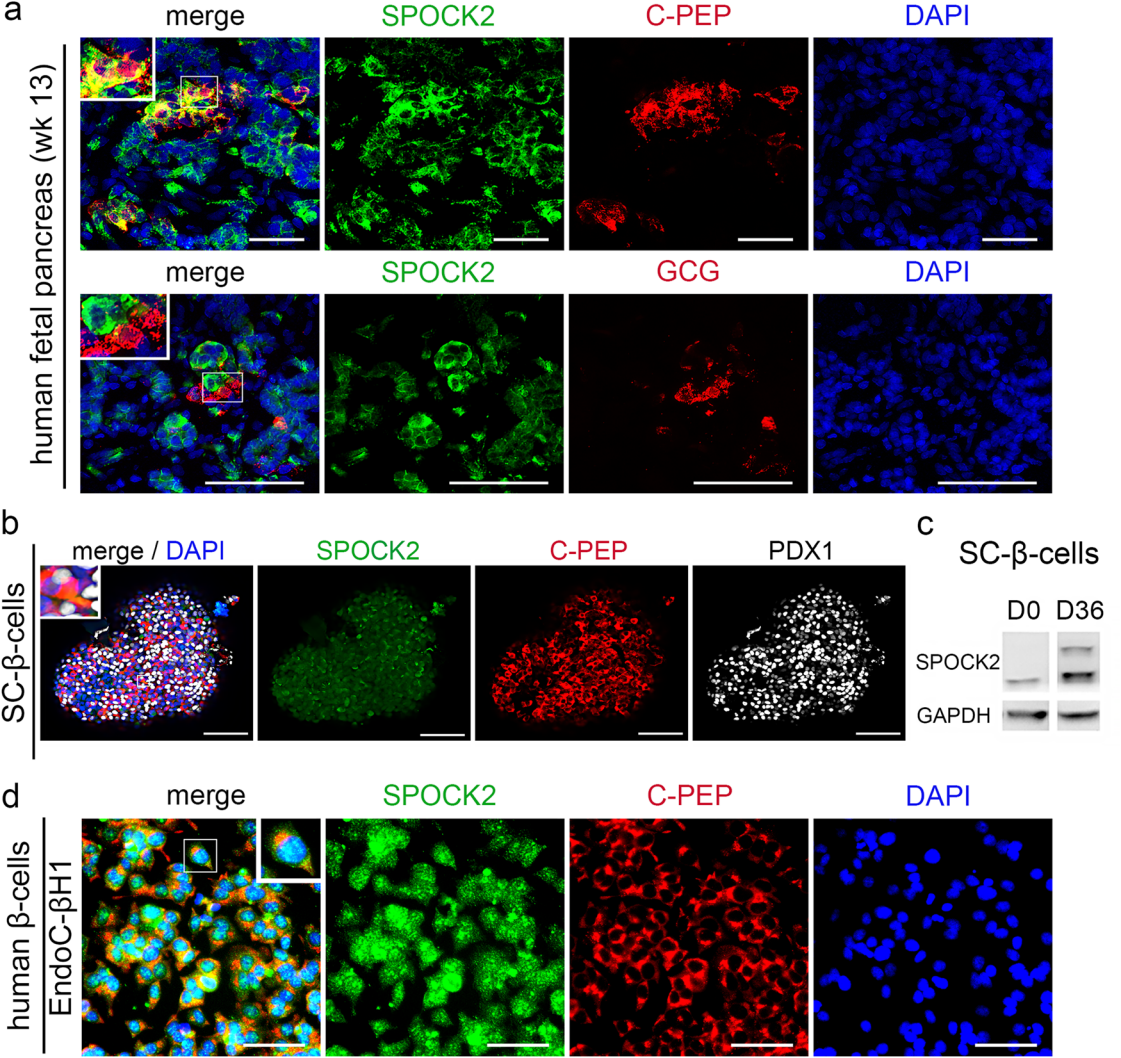
SPOCK2 is expressed in immature human β-cells. **a** Representative fluorescence microscopy images of the human fetal pancreas at week 13 (week 13) stained with antibodies against SPOCK2 (green) and C-peptide (C-PEP, red) to label early β-cells (upper panel). SPOCK2 (green) and glucagon (GCG, red) labeling of early α-cells (lower panel). Scale bars = 100 μm. DAPI (blue) was used to label the nuclei. *N* = 3 tissue samples. Examples of protein coexpression are presented in insets in the top corner of the merged images. **b** Representative confocal microscopy images of hPSC-derived β-cells (SC-β-cells) stained with antibodies against SPOCK2 (green), C-peptide (red) and PDX1 (white). DAPI (blue) was used to stain the nuclei. Scale bars = 100 μm. An example of protein coexpression is presented in the inset in the top left corner of the merged image. **c** Western blot analysis of SPOCK2 protein levels in undifferentiated hPSCs (Day 0, D0) and SC-β-cells (Day 36, D36). An antibody against GAPDH was used as a loading control. Two bands correspond to nonglycosylated and glycosylated SPOCK2 protein. **d** Representative fluorescence microscopy images of the EndoC-βH1 cells stained with antibodies against SPOCK2 (green) and C-peptide (red). DAPI (blue) was used to label the nuclei. Examples of protein coexpression are presented as insets in the top corner of the merged images. Scale bars = 100 μm.

In humans, EPs and early endocrine cells emerge between week 8 and week 13^59^. Immunofluorescence staining of the human pancreas at week 10 (Supplementary Fig. 1c) and week 13 (Fig. 1a and Supplementary Fig. 1d) revealed SPOCK2 expression in the developing pancreatic epithelium (Fig. 1a and Supplementary Fig. 1c, d). Cells positive for C-peptide (C-PEP), a cleavage product of proinsulin and a β-cell marker, were detected in the vicinity of the pancreatic epithelium at both timepoints (Fig. 1a and Supplementary Fig. 1c), which is consistent with β-cells arising from EPs delaminating from the epithelialium^60^. Most C-PEP^+^ cells were SPOCK2^+^ (Fig. 1a and Supplementary Fig. 1c), but not all SPOCK2^+^ cells were C-PEP^+^. We then investigated whether other hormone-producing cells in the pancreas coexpressed SPOCK2. At week 13, most glucagon (GCG)^-^expressing α-cells displayed no immunofluorescent signal for SPOCK2 (Fig. 1a). The very rare cases of marker overlap might correspond to late-stage EPs just starting to produce GCG. We also detected some coexpression of SPOCK2 and somatostatin (SST), a δ-cell marker (Supplementary Fig. 1d), which is intriguing, as β- and δ- cells both express pancreatic and duodenal homeobox 1 (PDX1), whereas other endocrine cell types do not. We concluded that SPOCK2 is expressed in most β-cells and some δ-cells but is barely detectable in α cells in the developing human pancreas.

**Fig. 2:**
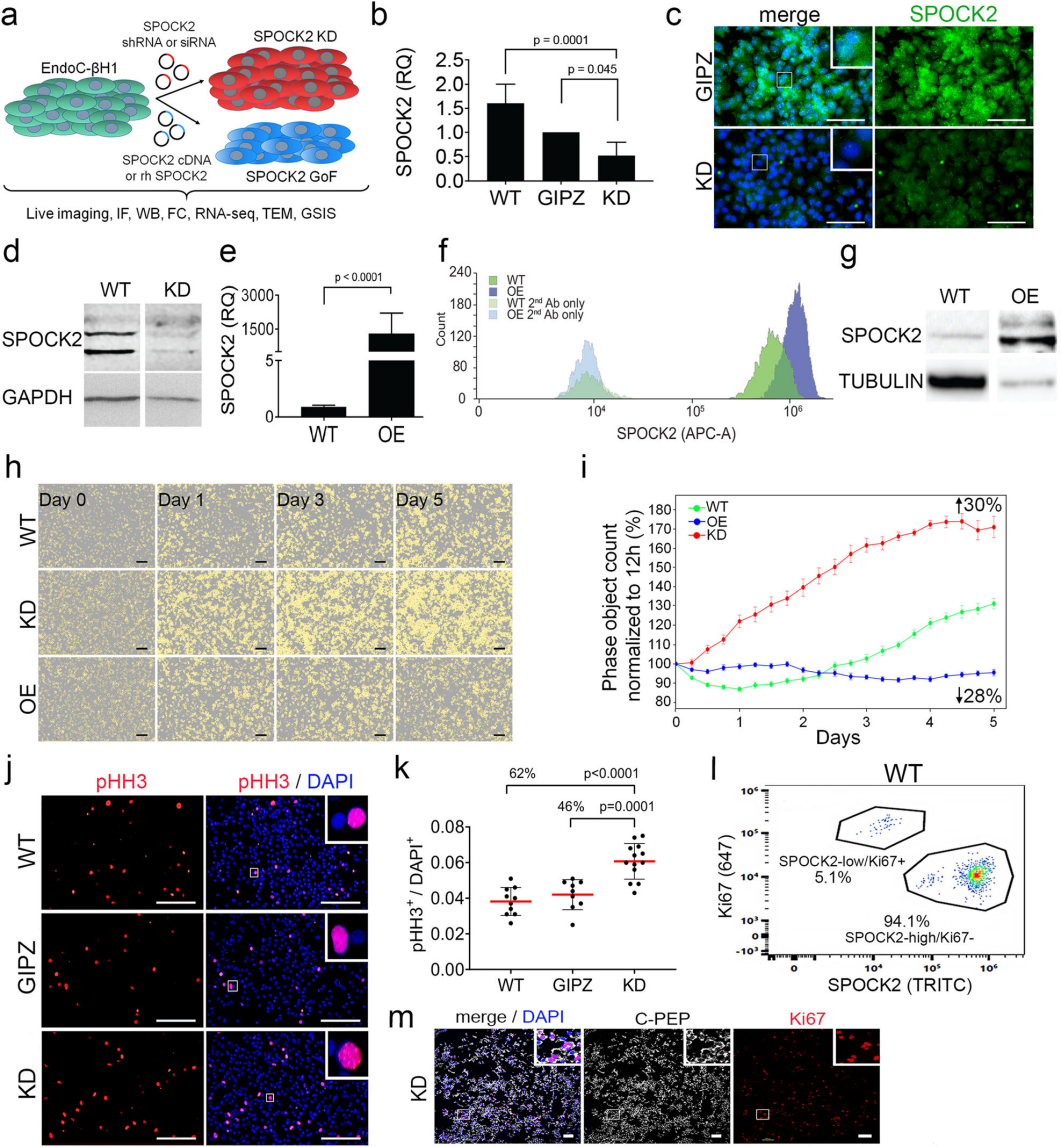
Bidirectional modulation of SPOCK2 expression controls early β-cell numbers in humans. **a** Schematic outline of the bidirectional modulation of SPOCK2 expression. To knock down (KD) SPOCK2 expression, shRNAs or siRNAs against SPOCK2 were applied to EndoC-βH1 cells. To increase SPOCK2 expression, transient overexpression of the SPOCK2 transcript or recombinant SPOCK2 (rh SPOCK2) was used. To characterize EndoC-βH1 cells with the changes in SPOCK2 expression, live imaging, RNA-seq, IF or GSIS was used. **b** RT‒qPCR analysis of *SPOCK2* mRNA levels in SPOCK2-KD, GIPZ-treated, and WT cells. One-way ANOVA for multiple comparisons was used to determine the *p* values. The data are presented as the means ± SDs. *N* = 3 biological replicates. **c** Representative fluorescence microscopy images of EndoC-βH1 SPOCK2-KD and GIPZ control cells stained with an antibody against SPOCK2 (green) showing lower levels of the SPOCK2 protein in SPOCK2-KD cells than in GIPZ control cells. Scale bars = 100 μm. DAPI (blue) was used to label the nuclei. **d** Western blot analysis of the SPOCK2 protein levels in EndoC-βH1 SPOCK2-KD and WT cells. An antibody against GAPDH was used as a loading control. **e** RT‒qPCR analysis of *SPOCK2* mRNA in SPOCK2 OE and WT cells. The *p* values were assessed by an unpaired two-tailed Student’s *t* test. The data are presented as the means ± SDs. *N* = 3 biological replicates. **f** FACS analysis of SPOCK2 protein levels (*x*-axis) in SPOCK2 OE (dark blue peak) and WT cells (dark green peak). SPOCK2 OE and WT cells incubated with secondary antibodies alone were used as controls, corresponding to the light blue and green peaks, respectively. **g** Higher SPOCK2 protein levels were detected in EndoC-βH1 SPOCK2 OE cells than in WT cells, as demonstrated by western blotting. An antibody against tubulin was used as a loading control. **h** EndoC-βH1 SPOCK2-KD, SPOCK2-OE, and WT cells were monitored via live cell imaging over a five-day culture period. Representative bright-field images (cells marked with a yellow mask) of EndoC-βH1 WT, SPOCK2-KD, and SPOCK2-OE cells on Days 0, 1, 3, and 5 are shown. Scale bars = 100 μm. **i** Quantification of phase object counts over a five-day time course normalized against the 12 h time point, showing the difference in total cell number between SPOCK2-KD (red) and SPOCK2-OE (blue) cells relative to WT cells (green). *N* = 3 biological replicates. **j** Representative fluorescence microscopy images of EndoC-βH1 WT (top), GIPZ control (middle), and SPOCK2 KD (bottom) cells stained with an antibody against the proliferation marker pHH3 (red). Examples of pHH3 nuclear detection with DAPI (blue) staining of the nuclei are presented as insets in the top right corners of the merged images. Scale bars = 100 μm. **k** Quantification of pHH3^+^ cells in EndoC-βH1 WT, GIPZ control, and SPOCK2 KD cells, shown as the ratio of pHH3^+^ to total live cells (DAPI^+^). The larger number of SPOCK2-KD cells expressing pHH3 than WT and GIPZ control cells expressing this protein is shown as a %. One-way ANOVA for multiple comparisons was used to determine the *p* values shown on the graph. The data are presented as the means ± SDs. *N* = 9–12 biological replicates. **l** Spectral flow cytometry analysis of SPOCK2 and Ki67 protein expression in WT EndoC-βH1 cells, showing a population of proliferating cells with low SPOCK2 levels (SPOCK2-low/Ki67 + ) and a population of nonproliferating cells with higher SPOCK2 levels (SPOCK2-high/Ki67-). **m** Coexpression of Ki67 (red) and C-PEP (white) in SPOCK2-KD EndoC-βH1 cells. Scale bars = 100 μm. DAPI (blue) was used to stain the nuclei. Enlarged views are shown in the insets in the top corner of the images.

To study the role of SPOCK2 in immature human β-cells, we analyzed SC-β-cells and found that SPOCK2 was expressed in both C-PEP^+^ and C-PEP^-^ cells at the final stage of hPSC differentiation (Fig. 1b and Supplementary Fig. 1e), which was consistent with the results of scRNA-seq (Supplementary Fig. 1a, b) and fetal tissue staining (Fig. 1a and Supplementary Fig. 1c, d). Western blot analysis confirmed the presence of the SPOCK2 protein in SC-β-cells on Day 36, revealing the presence of nonglycosylated and glycosylated SPOCK2^61^ (Fig. 1c). To minimize the impact of variable efficiency of β-cell formation from hPSCs, we employed an additional system, the EndoC-βH1 cell line, which in some respects resembles human fetal β-cells. The EndoC-βH1 line was obtained via SV40LT immortalization of the human fetal pancreas (weeks 7–11), which was then allowed to differentiate into β-cells^34^. The EndoC-βH1 cell proliferation rate was 2–4% for fetal β-cells (^34^Fig. 2h-m), and these cells expressed C-PEP, a panendocrine marker, chromogranin A (CHGA), and the key β-cell transcription factor PDX1 (Supplementary Fig. 1f). We compared C-PEP expression between early- and late-passage cells separated by two years and found no significant β-cell dedifferentiation (Supplementary Fig. 1g). Finally, immunofluorescence staining confirmed SPOCK2 expression in C-PEP^+^ EndoC-βH1 cells (Fig. 1d). Thus, we deemed SC-β-cells and the EndoC-βH1 cell line suitable for studies on the role of SPOCK2 in human fetal-like β-cells.

### Bidirectional dysregulation of SPOCK2 expression affects human β-cell proliferation

To investigate the effect of SPOCK2 levels on human β-cells, we used lentiviral-based delivery of shRNA or siRNA transfection to achieve *SPOCK2* knockdown (KD), and for the opposite effect, we transiently overexpressed (OE) *SPOCK2* cDNA or applied recombinant SPOCK2 protein (rh SPOCK2) to EndoC-βH1 cells (Fig. 2a). We confirmed the downregulation and upregulation of SPOCK2 at both the mRNA and protein levels in KD and OE cells, respectively (Fig. 2b–g and Supplementary Fig. 2a, b). Decreased SPOCK2 expression was maintained over multiple passages in KD EndoC-βH1 cells (Supplementary Fig. 2c). During the culture of KD and OE EndoC-βH1 cells, we observed differences in both total cell number and Green fluorescent protein (GFP^+^) cell number, with GFP used as a readout for transgene delivery. Over five days of culture, the total cell numbers were 30% greater and 28% lower for KD and OE, respectively, than for nontransfected cells (Fig. 2h, i and Video Supplementary 1). Additionally, during eight days of culture, the number of GFP+ cells increased by 68% in KD cells and decreased by 56% in OE cells (Supplementary Fig. 2d, e). The increased expression of the proliferation markers phospho-histone 3 (pHH3, Fig. 2j, k) and Ki67 (Supplementary Fig. 2f, g) in KD EndoC-βH1 cells corroborated these observations. Interestingly, in WT EndoC-βH1 cells, we distinguished two populations on the basis of the protein expression of SPOCK2 and Ki67. Specifically, 5.1% of the cells presented low SPOCK2 expression and were positive for Ki67, and 94.1% of the total cells were nonproliferating (Ki67- with high SPOCK2 expression). These findings suggest that SPOCK2 expression is inversely correlated with Ki67 expression at the single-cell level (Fig. 2l). Furthermore, we confirmed that the proliferating Ki67^+^ KD cells were C-PEP^+^, suggesting that SPOCK2 KD cells did not undergo dedifferentiation (Fig. 2m**)**. Finally, in parallel, we downregulated SPOCK2 in EndoC-βH1 cells with siRNA, leading to a 50% decrease in protein abundance (Supplementary Fig. 2h, i) and a 30% increase in β-cell number compared with those in untreated and scramble-treated cells (Supplementary Fig. 2j).

### SPOCK2 controls the proliferation of SC-β-cells

**Fig. 3:**
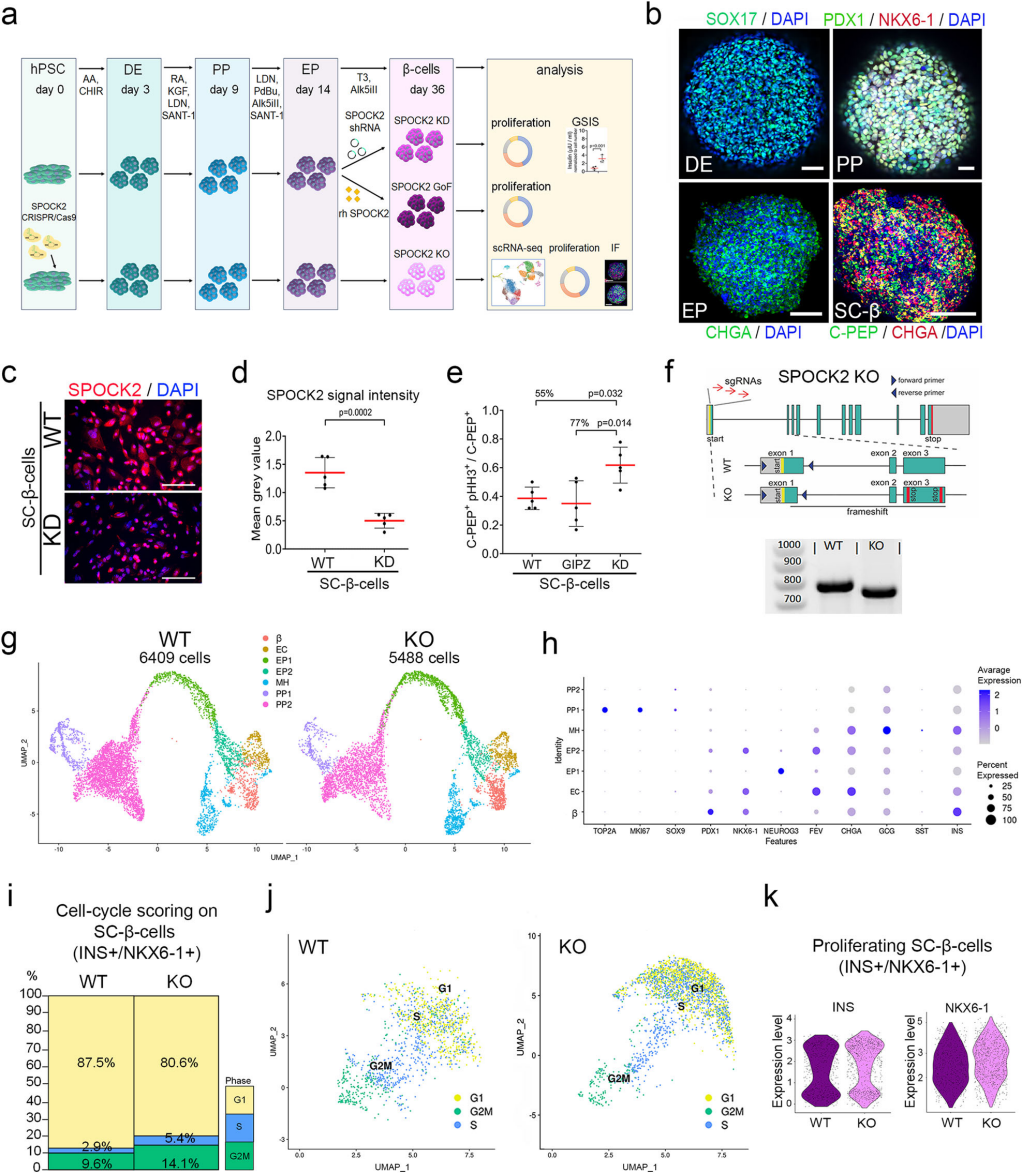
SPOCK2 regulates early β-cell proliferation. **a** Schematic outline of the experimental design used to study the role of SPOCK2 in SC-β-cells. SPOCK2-KD, shRNAs against SPOCK2 or control shRNAs were added to the cells at the EP stage. In contrast, cells at the EP stage were treated with recombinant SPOCK2 (rh SPOCK2) protein. For SPOCK2 knockout (KO), CRISPR/Cas9 and clonal selection were used to delete the SPOCK2 gene in hPSCs, followed by differentiation into β-cells. Early hPSC-derived β-cells were subjected to proliferation analysis, IF, GSIS or scRNA-seq. DE: definitive endoderm, PP: pancreatic progenitors, EP: endocrine progenitors **b** Representative fluorescence microscopy images of cells during hPSC differentiation into β-cells stained with antibodies against markers of consecutive differentiation stages: definitive endoderm (DE) cells expressing SOX17 (green); pancreatic progenitors (PP) co expressing PDX1 (green) and NKX6-1 (red); endocrine progenitors (EP) stained for CHGA (green), SC-β-cells stained for C-PEP (green) and CHGA (red). DAPI (blue) was used to stain the nuclei. Scale bars = 100 μm. **c** Representative fluorescence microscopy images of WT and SPOCK2-KD SC-β-cells stained with an antibody against SPOCK2 (red). DAPI (blue) was used to stain the nuclei. Scale bars = 100 μm. **d** Quantification of normalized fluorescence signals from Fig. 3C. *N* = 5 biological replicates. **e** Quantification of proliferating SC-β-cells. The ratio of C-PEP^+^/pHH3^+^ cells to C-PEP^+^ WT, GIPZ, or SPOCK2-KD cells. The larger number of SPOCK2-KD SC-β-cells expressing pHH3 than control cells is shown as a percentage. One-way ANOVA for multiple comparisons was used to determine the *p* values. The data are presented as the means ± SDs. *N* = 5 biological replicates. **f** SPOCK2-KO hPSC line generation. The top 3 sgRNAs targeting SPOCK2 exon 1 were cotransfected into hPSC-iCas9, and clones with frameshifts and introduction of the stop codon in exon 3 were selected for further analysis. Bottom: Agarose gel electrophoresis of DNA from selected single-cell clones of the SPOCK2-/- (KO) line compared with the WT line. **g** UMAP representation of single-cell transcriptomes of 5,500 SPOCK2 KO and 6,400 WT SC-β single cells grouped via graph-based clustering, with 7 distinct clusters: PP1—proliferating pancreatic progenitors; PP2—pancreatic progenitors; EP1—early endocrine progenitors; EP2—late endocrine progenitors; EC—enterochromaffin; MH—multihormonal cells; and β—immature β-cells. **h** Bubble plot of unique marker transcripts (shown on the *x*-axis) enriched in pancreatic progenitors (PPs), endocrine progenitors (EPs), or endocrine cell clusters (shown on the *y*-axis). The bubble size represents the percentage of cells expressing a gene, whereas the color intensity indicates the average scaled gene expression in a sample. **i** Cell cycle scoring of SPOCK2 KO and WT SC-β-cell scRNA-seq data showing the proportion (%) of SPOCK2 KO and WT SC-β-cells (defined as INS^+^/NKX6.1^+^) in phases G1, S and G2M. **j** UMAP plot showing the distribution of WT (left) and SPOCK2-KO (right) SC-β-cells in different phases of the cell cycle. The cells in phase G1 are red, those in G2M are green, and those in S are blue. **k** Violin plot showing INS expression levels in SPOCK2 KO and WT SC-β-cells in different cell cycle phases.

We next evaluated the effects of SPOCK2 on SC-β-cell proliferation. We employed two- or three-dimensional (2D and 3D) pancreatic differentiation protocols^2^, which guided hPSCs (HUES8, HUES3 or H1 human embryonic stem cell lines) into β-cells in a stepwise manner (Fig. 3a). We confirmed successful progression by assessing the expression of stage-specific markers: SRY-box transcription factor 17 (SOX17) for definitive endoderm (DE), PDX1 and NKX6.1 for pancreatic progenitors (PP), CHGA for EP, and C-PEP for the β-cell stage. At the DE stage, ~90% of the cells expressed SOX17; at the PP stage, ~80% of the cells were PDX1^+^, ~60% were NKX6-1^+^ and PDX1^+^; and at the EP stage, ~ 50% were CHGA^+^. At the β-cell stage (Day 36), 50% to 70% of the cells were C-PEP^+^ or CHGA^+^ (Fig. 3b), indicating the successful generation of β-cells from hPSCs. We next investigated whether the rh SPOCK2 protein inhibits the proliferation of SC-β-cells. We first enriched SC-β-cells via an antibody against the CD49a antigen (also called integrin subunit alpha 1, ITGA1)^6^ and transmembrane 4 L six family member 4 (TM4SF4)^62^, which are human β-cell and α-cell surface markers, respectively (Supplementary Fig. 3a). The sorted CD49a^+^/TM4SF4^-^ SC-β-cells were treated with 100 ng/ml or 250 ng/ml rh SPOCK2, which caused a 44% decrease in the number of pHH3^+^ cells at higher concentrations (Supplementary Fig. 3b). Concurrently, treatment of WT EndoC-βH1 cells with rh SPOCK2 for seven days resulted in a dose-dependent decrease in pHH3^+^ cells (Supplementary Fig. 3c, d).

We next employed a lentiviral system to deliver an anti-*SPOCK2* shRNA or an empty GIPZ plasmid, both containing the GFP sequence, leading to transduction of ~70% of the cells with anti-*SPOCK2* shRNA (GFP^+^ cells, Fig. 3c and Supplementary Fig. 3e) and ~60% downregulation of the SPOCK2 protein (Fig. 3d) at the SC-β-cell stage. We noted 55% and 77% increases in C-PEP^+^/pHH3^+^ for SPOCK2-KD, relative to WT or GIPZ, respectively (Fig. 3e). However, the proliferation of SC-β-cells was low, at ~0.6% of the total number of cells.

To overcome the limitations of the shRNA approach, we next employed CRISPR/Cas9 to generate an hPSC clonal line with SPOCK2 knockout (KO), which was subsequently differentiated into SC-β-cells (Fig. 3a, f). We used three sgRNAs to target exon 1 of *SPOCK2*, which encodes the acidic region, the unique domain among the SPARC family^63^ **(**Fig. 3f). DNA sequencing (data not shown) and genomic PCR with primers flanking the targeted region (Fig. 3f) confirmed the deletion and premature stop codon in exon 3 of the *SPOCK2* gene. The loss of SPOCK2 protein expression in SPOCK2-KO cells was also confirmed by immunofluorescence staining at the early β-cell stage (Day 21 of differentiation) **(**Supplementary Fig. 3f). To investigate the proliferation of SPOCK2-KO SC-β-cells, we transcriptionally profiled ~6400 WT and ~5500 KO cells via scRNA-seq (10x Genomics, Fig. 3g). We performed filtering, normalization, variable gene identification, linear regression for batch, and PCA with the R package Seurat^55^ and identified 7 distinct populations (Fig. 3g). On the basis of the presence of unique markers, we named the populations PP1 (proliferating PP), PP2, EP1, EP2, enterochromaffin (EC), multihormonal (MH) and immature β-cells (Fig. 3h). We then used cell cycle scoring within the Seurat package to identify proliferating β-cells^56^. Compared with 12% of WT SC-β-cells, 19% of SPOCK2-KO cells, which expressed INS and NKX6.1, were in the S and G2/M phases of the cell cycle (Fig. 3i–k). Overall, we consistently observed that SPOCK2 inhibits the proliferation of immature human β-cells. We used multiple approaches to manipulate SPOCK2 expression and three independent hPSC lines, HUES3, HUES8 and H1, to derive β-cells, indicating that the effect of SPOCK2 on human β-cell proliferation is not restricted to EndoC-βH1 cells or a particular hPSC line.

### SPOCK2 downregulation improves insulin secretion in human fetal-like β-cells

**Fig. 4:**
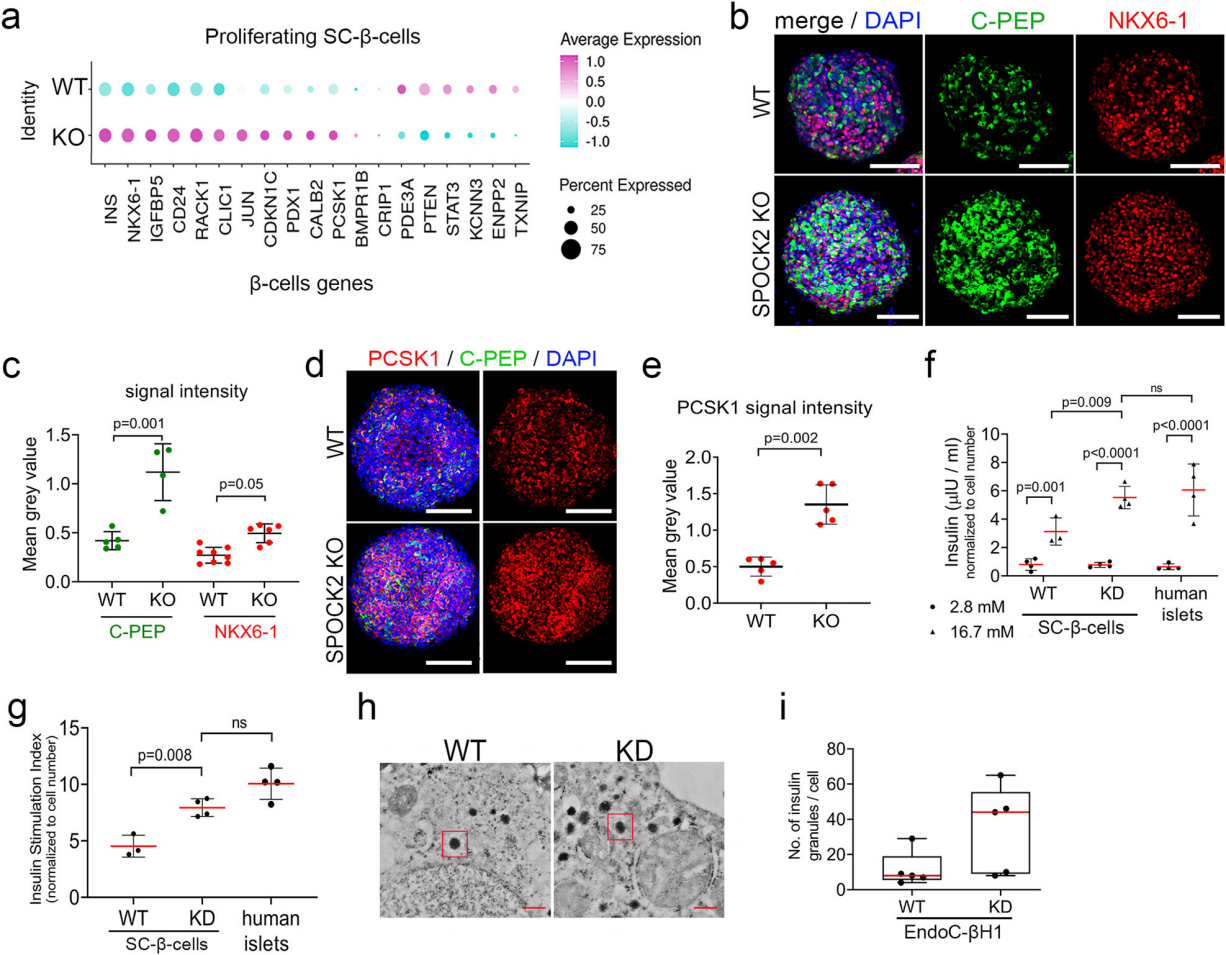
SPOCK2 improves immature β-cell function. **a** Bubble plot showing the relative expression of selected β-cell-associated genes in SPOCK2-KO and WT SC-β-cells. The bubble size represents the percentage of cells expressing a gene, whereas the color intensity indicates the average scaled gene expression in a sample. Pink signifies upregulation, and turquoise indicates downregulation of gene expression. **b** Representative confocal microscopy images of SPOCK2 KO and WT SC-β-cells stained with antibodies against C-PEP (green) and NKX6-1 (red). DAPI (blue) was used to stain the nuclei. Scale bars = 100 μm. **c** Quantification of fluorescence signals from C-PEP and NKX6-1 staining (Fig. 4B). Higher protein levels were detected in SPOCK2-KO SC-β-cells than in WT SC-β-cells, with *p* values determined via Student’s *t* test and presented in the graph. The data represent the means ± SDs from 4 biological replicates. **d** Representative confocal microscopy images of SPOCK2 KO and WT SC-β-cells stained with antibodies against C-PEP (green) and PCSK1 (red). DAPI (blue) was used to stain the nuclei. Scale bars = 100 μm. **e** Quantification of fluorescence signals from PCSK1-stained cells (Fig. 4D). Student’s *t* test was used to determine the *p* values shown on the graph. The data are presented as the means ± SDs. *N* = 5 biological replicates. **f** Insulin secretion from WT or SPOCK2-KD SC-β-cells and human primary islets challenged with low glucose (2.8 mM) or high glucose (16.7 mM) was normalized to the total cell number. One-way ANOVA for multiple comparisons was used to determine the *p* values shown on the graph. The data are presented as the means ± SDs. *N* = 4 biological replicates. **g** Insulin stimulation index of WT and SPOCK2-KD SC-β-cells compared with that of human primary islets, calculated as the ratio of insulin secretion in response to 16.7 mM vs. 2.8 mM glucose. One-way ANOVA for multiple comparisons was used to determine the *p* values shown on the graph. The data are presented as the means ± SDs. *N* = 4 biological replicates. **h** Electron microscopy images of WT and SPOCK2-KD EndoC-βH1 cells, with representative insulin granules shown in red; scale bar = 500 nm. **i** Box and whisker plot of the number of insulin granules per cell. The thick horizontal line indicates the median. *N* = 5 WT and SPOCK2-KD EndoC-βH1 cells.

Interestingly, the proliferating SPOCK2 KO SC-β-cells expressed higher *INS* and *NKX6-1* levels (Figs. 3k and 4a), suggesting further maturation. This prompted us to investigate the cohort of genes associated with β-cell function. We detected increased expression of *PDX1*, *JUN*, and Proprotein convertase 1 *(PCSK1)* and decreased expression of *TXNIP*, suggesting that SPOCK2 KO β-cells are more functional (Fig. 4a). We confirmed elevated C-PEP, NKX6-1 and PCSK1 protein levels in SPOCK2-KO SC-β-cells (Fig. 4b–e). Similarly, bulk RNA-seq of SPOCK2-KD EndoC-βH1 cells revealed increased expression of genes known to positively regulate insulin synthesis, including *INS*, paired box 4 (*PAX4*), MAF BZIP transcription factor B (*MAFB)*, *NKX6-1*, and *RAB3A* (Supplementary Fig. 4a), and immunofluorescence confirmed increased C-PEP and CHGA expression (Supplementary Fig. 4b, c). Therefore, we next performed a GSIS assay to evaluate the function of SPOCK2-deficient SC-β and EndoC-βH1 cells. Control and SPOCK2-KD cells presented minimal INS secretion in response to 2.8 mM glucose, but when exposed to 16.7 mM glucose, SPOCK2-KD SC-β-cells secreted an average of 5.5 µlU/ml INS, representing an increase of 85% (or 100% in the case of EndoC-βH1-KD cells) (Fig. 4f and Supplementary Fig. 4d). The INS stimulation index (SI), defined as the ratio of high-to-low glucose-induced INS secretion, was significantly greater in SPOCK2-KD SC-β-cells and EndoC-βH1 cells than in their WT counterparts (Fig. 4g and Supplementary Fig. 4e). Notably, INS secretion in response to 16.7 mM glucose and the INS SI of SPOCK2-KD SC-β-cells was comparable to that of human primary islets (Fig. 4f, g). Finally, transmission electron microscopy revealed more insulin granules in SPOCK2-KD cells than in WT EndoC-βH1 cells (Fig. 4h, i). Collectively, these data suggest that SPOCK2 deficiency in fetal-like human β-cells increases the expression of a cohort of genes associated with INS secretion, leading to enhanced function.

### MMP2, the SPOCK2 effector, and WNT synergistically regulate fetal-like β-cell proliferation in vitro

**Fig. 5:**
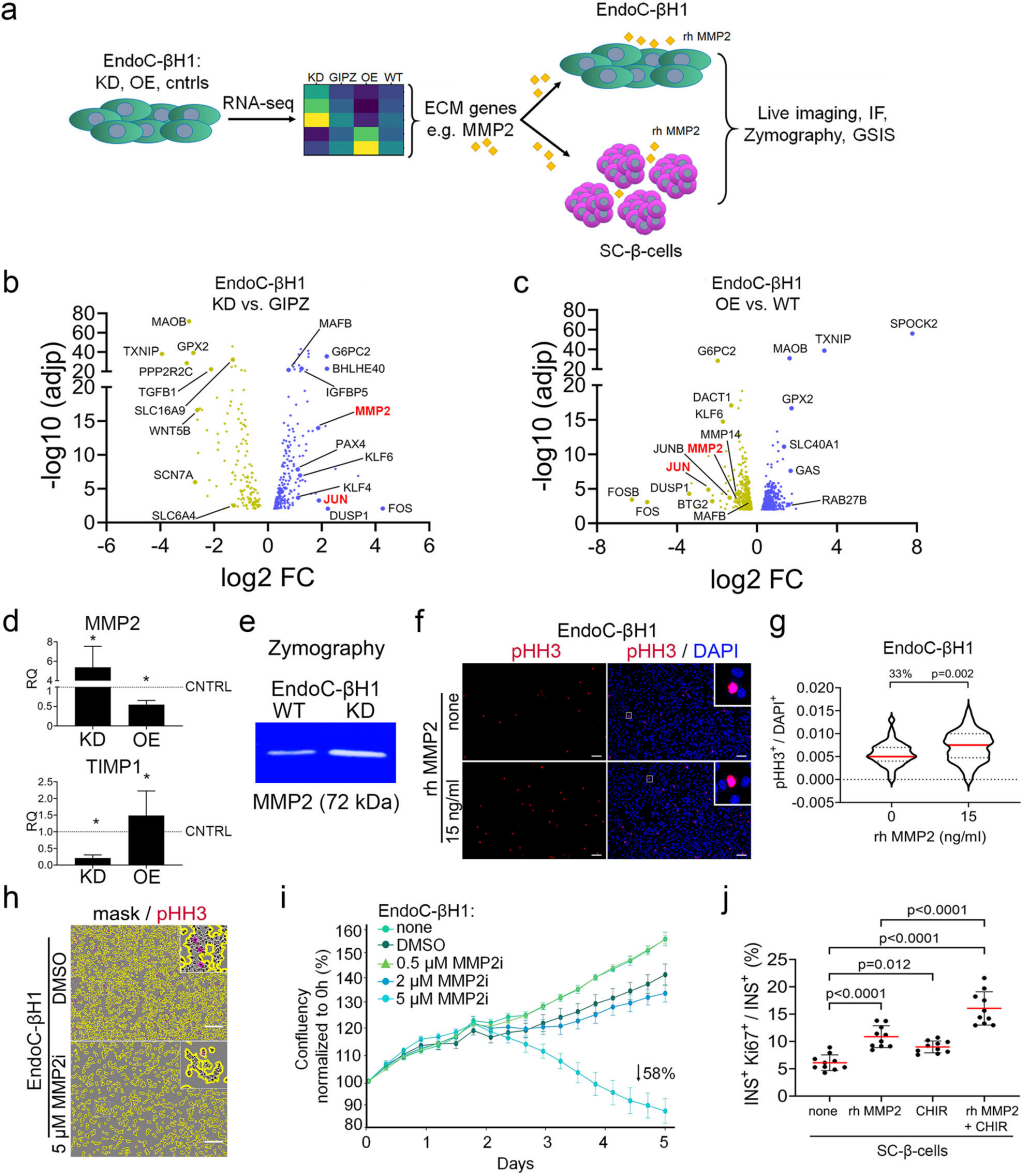
SPOCK2-mediated MMP2 activation increases EndoC-βH1 and SC-β-cell proliferation. **a** Schematic outline of the experimental design used to study the role of candidate genes in EndoC-βH1 and SC-β-cells. On the basis of the RNA-seq data from the SPOCK2-KD and SPOCK2-OE strains, MMP2 was selected as a candidate gene. EndoC-βH1 or SC-β-cells were treated with recombinant MMP2 (rh MMP2) and further subjected to live imaging, zymography, IF or GSIS. **b** Volcano plot of DEGs between EndoC-βH1 SPOCK2-KD and GIPZ control cells, with selected genes marked. The violet dots correspond to upregulated (-log10 (adjp) ≥2 and log2FC ≥ 0.5), and the yellow dots correspond to downregulated (-log10 (adjp) ≥2 and log2FC ≤ –0.05) genes. **c** Volcano plot depicting DEGs between EndoC-βH1 SPOCK2 OE and WT cells, with selected genes marked. The violet dots correspond to upregulated (-log10 (adjp) ≥2 and log2FC ≥ 0.5), and the yellow dots correspond to downregulated (-log10 (adjp) ≥2 and log2FC ≤ -0.05) genes. **d** RT‒qPCR results showing the expression of *MMP2* and the MMP inhibitor *TIMP1*. Student’s t test was used to determine significance. The data are presented as the means ± SDs. * ≤0.05. *N* = 3 biological replicates. **e** Zymography showing increased MMP2 activity in SPOCK2-KD EndoC-βH1 cells. **f** Representative fluorescence microscopy images of EndoC-βH1 WT cells treated for seven days with 15 ng/mL rh MMP2 protein and stained with an antibody against pHH3 (red). Untreated WT cells served as a control (none). DAPI (blue) was used to stain the nuclei. Scale bars = 100 μm. **g** Quantification of pHH3^+^ EndoC-βH1 WT cells, shown as the ratio of pHH3^+^ cells to the total number of DAPI^+^ cells. The greater percentage of pHH3^+^ EndoC-βH1 after treatment with 15 ng/mL rh MMP2 is shown as a %. One-way ANOVA for multiple comparisons was used to determine the *p* value shown on the graph. The data are presented as the means ± SDs. *N* = 5 biological replicates. **h** Representative bright-field images of cells marked with a yellow mask and stained with an antibody against pHH3 (red). Compared with DMSO-treated cells, EndoC-βH1 WT cells treated with 5 μM MMP2i for five days presented fewer total cells (confluent) and pHH3^+^ cells. Scale bars = 100 μm. **i** Quantification of the confluence of EndoC-βH1 cells treated with different concentrations of MMP2i for five days normalized to 0 h. *N* = 3 biological replicates. **j** Quantification of proliferating SC-β-cells (INS^+^/Ki67^+^) via flow cytometry after MMP2 and CHIR99021 treatment; the results are presented as a percentage of the control values. One-way ANOVA for multiple comparisons was used to determine the *p* value shown on the graph. The data are presented as the means ± SDs. *N* = 3 biological replicates.

Given the limitations of genetically manipulating SPOCK2 expression and the lack of publicly available SPOCK2 inhibitors, we searched for potential SPOCK2 effectors (Fig. 5a). RNA-seq of KD, OE, WT and GIPZ EndoC-βH1 revealed dysregulated expression of hundreds of genes, and we initially focused on secreted or ECM proteins (Fig. 5a–c and Supplementary Table 1). One of the genes with the greatest difference, with a log2-fold change (FC) of 1.87 and adjusted *p* value (adjp) of 1.1 × 10^–14,^ was the ECM protein *MMP2* **(**Fig. 5b, c). Notably, the ECM was one of the top enriched KEGG and Wiki pathways in KD (Supplementary Fig. 7b). Using qRT‒PCR, we confirmed that *MMP2* expression was elevated in SPOCK2-KD cells and decreased in SPOCK2-OE cells (Fig. 5d). A similar expression profile was observed for the MMP2-positive regulator MMP14^64,65^ (Supplementary Table 1), whereas TIMP metallopeptidase inhibitor 1 *(TIMP1)*, a potential MMP2 inhibitor^66^, exhibited the opposite expression pattern (Fig. 5d). SPOCK2 might regulate MMP2 levels, as it was suggested that SPOCK2 sequesters MMP2^63,67,68^. Given that MMP2 can be used in in vitro culture as a recombinant protein, we chose to scrutinize MMP2 as a potential downstream effector of SPOCK2. We first tested MMP2 activity in SPOCK2-KD EndoC-βH1 cells. Zymography revealed elevated proteolytic MMP2 activity, as indicated by gelatin digestion, in supernatants from the SPOCK2-KD cells (Fig. 5e). Treatment of WT EndoC-βH1 cells with rh MMP2 increased the pHH3^+^ cell number (Fig. 5f, g); conversely, the MMP2 inhibitor (MMP2i) caused a dose-dependent decrease in cell confluency and the pHH3^+^ cell number (Fig. 5h, i and Supplementary Fig. 5a, b).

We next compared SPOCK2 KD to other mitogens, including CHIR99021, a canonical Wnt pathway activator^69^. CHIR99021 treatment increased Ki67 expression in WT EndoC-βH1 cells, similar to SPOCK2 KD **(**Supplementary Fig. 5c, d). When SPOCK2 KD and CHIR99021 were combined, an additive effect was measured, with a 44% increase over CHIR99021- and an 80% increase in Ki67^+^ cells over DMSO-treated WT cells **(**Supplementary Fig. 5c, d). Treatment of SC-β-cells with 15 ng/ml MMP2 caused a 66% increase in the number of Ki67^+^/INS^+^ cells (6% vs. 10% of total cells), whereas CHIR99021 + MMP2 increased SC-β-cell proliferation by 300% (Fig. 5j). WS6 (an inhibitor of Erb3 binding protein-1 and the IκB kinase pathway) or GW788388 (a TGF-β pathway inhibitor) also had mitogenic effects, but the effect was weaker than that of MMP2 + CHIR99021 (Supplementary Fig. 5e, f). Interestingly, harmine, a DYRK1A inhibitor and a potent adult β-cell mitogen do not induce fetal β-cell replication^22,69^. We also observed no positive effect of harmine, alone or with WS6, on immature β-cell proliferation (Supplementary Fig. 5e, f), suggesting that signals regulating immature β-cell proliferation might differ from those in adults.

### Long-term treatment of SC-β-cells with MMP2 leads to β-cell expansion and maturation

**Fig. 6:**
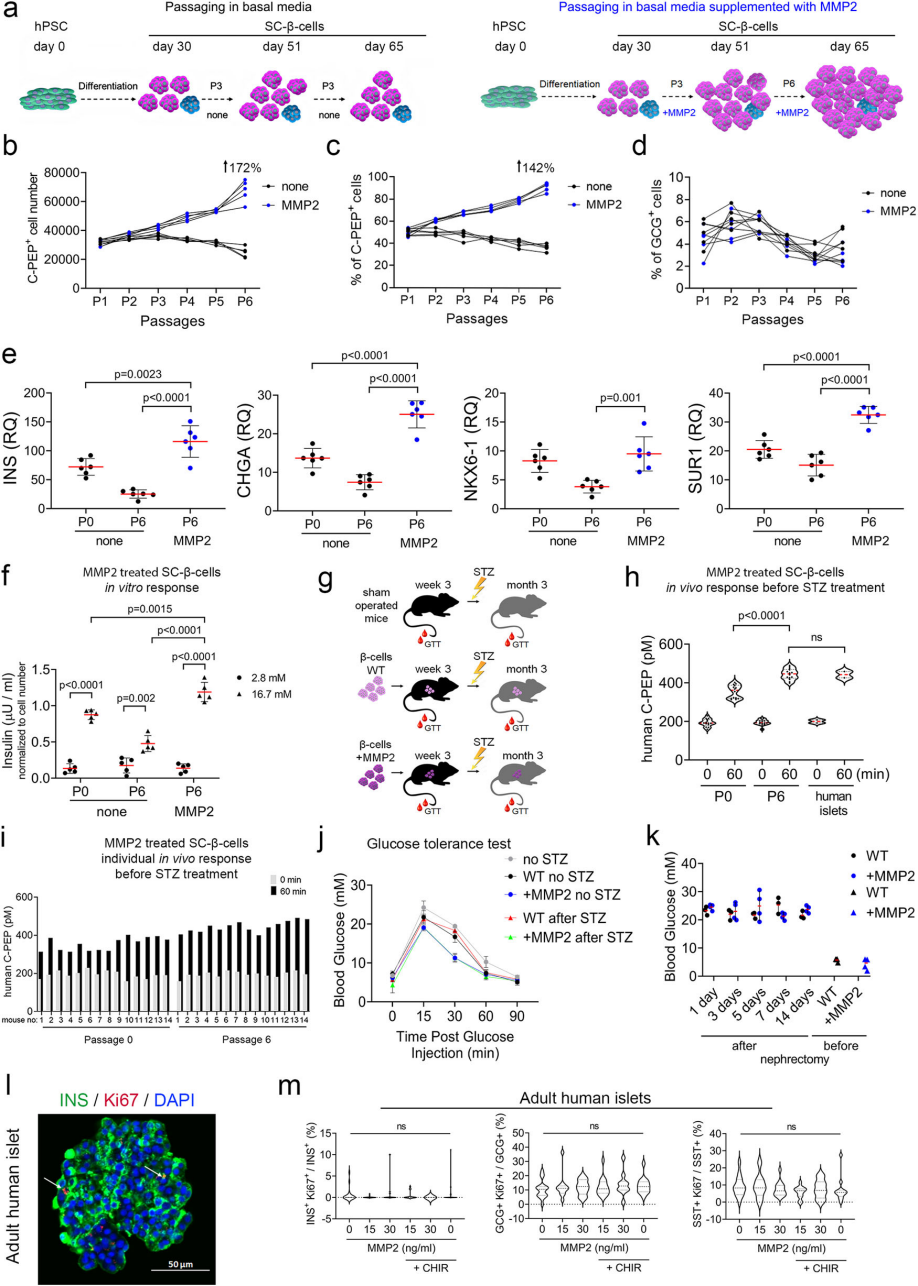
MMP2 induces human fetal β-cell proliferation, leading to β-cell expansion and increased insulin secretion in vitro and in vivo. **a** Schematic representation of the experimental design used to assess the long-term expansion of SC-β-cells with MMP2. hPSCs were differentiated into β-cells and subsequently cultured and passaged in either basal medium or medium supplemented with rh MMP2. **b** Quantification of the number of C-PEP^+^ SC-β-cells after 5 passages in the presence of rh MMP2. **c** Quantification of the percentage of C-PEP^+^ SC-β-cells after 5 passages in the presence of rh MMP2. **d** Constitutive passaging (5 passages) of hPSC-derived endocrine cells (Day 36) in the presence of rh MMP2 did not increase the percentage of GCG+ cells compared with that of the untreated control. **e** qRT‒PCR analysis of the expression levels of β-cell-specific genes, including *INS*, *CHGA*, *NKX6-1* and *SUR1*, in nonpassaged and passaged SC-β-cells in the presence of MMP2. The *p* values were assessed by an unpaired two-tailed Student’s *t* test. The data are presented as the means ± SDs. *N* = 3 biological replicates. **f** Insulin secretion in SC-β-cells, both nonpassaged and passaged in the presence of MMP2 and challenged with low glucose (2.8 mM) or high glucose (16.7 mM), normalized to the total cell number. One-way ANOVA for multiple comparisons was used to determine the *p* values shown on the graph. The data are presented as the means ± SDs. *N* = 3 biological replicates. **g** Scheme of the experimental design used to test the function of untreated and MMP2-treated SC-β-cells in vivo. SC-β-cells, either unpassaged or passaged in the presence of MMP2, were transplanted under the kidney capsules of immunocompromised SCID-Beige mice. A control group of mice underwent sham surgery without SC-β-cell transplantation. Three weeks after transplantation, a glucose tolerance test (GTT) was conducted. After three months, the mice were injected with streptozotocin (STZ) to ablate murine β-cells, followed by a second GTT assessment. **h** ELISA measurements of human insulin in the serum of mice transplanted with comparable numbers of SC-β-cells, treated short- and long-term with rh MMP2, or with primary human islets (~700 IEQ). Measurements were taken before (white bars) and 60 min after (black bars) glucose injection in the mice 3 weeks after transplantation. A one-way ANOVA for multiple comparisons was used to determine the *p* values shown on the graph. The data are presented as the means ± SDs. *N* = 14 biological replicates. **i** ELISA measurements of human insulin in the serum of individual mice transplanted with comparable numbers of rh MMP2-treated SC-β-cells in the short and long term. Measurements were taken before (white bars) and 60 min after (black bars) glucose injection in the mice 3 weeks after transplantation. **j** Blood glucose levels in mice 3 weeks after transplantation with non-STZ-induced SC-β-cells (WT, no STZ) or rh MMP2-treated (+MMP2, no STZ) and in the same mice 3 months after the use of STZ to induce diabetes (WT after STZ or +MMP2 after STZ). As controls, sham-operated mice before (sham, no STZ) and after STZ (sham, after STZ) injections were used. The mice were fasted for 16 h, and blood glucose was subsequently measured before (0 min) and 15, 30, 60 and 90 min after glucose injection. The data are presented as the means ± SDs. *N* = 4 mice. The *p* value was determined using a *t* test. **k** Blood glucose levels in diabetic (STZ-induced) mice following the removal of kidney grafts containing rh MMP2-treated (+MMP2) or untreated (WT) SC-β-cells. Fasting blood glucose was measured at 1, 3-, 5-, 7- and 14-days post nephrectomy (*n* = 3–4 mice per group). The corresponding STZ-treated mice before graft removal were used as controls (*n* = 3–4 mice). The data are presented as the means ± SDs. The *p* values were determined via a *t* test. **l** Representative image of human islets stained with antibodies against Ki67 (red) and insulin (INS, green). Nuclei were stained with DAPI (blue). Scale bar = 50 μM. **m** Quantification of Ki67 + /INS+ (left), Ki67 + /GCG+ (middle) or Ki67 + /SST+ (right) cells after MMP2, CHIR99021, MMP2 + CHIR99021, or vehicle (DMSO) treatment of human primary islets for 7 days. One-way ANOVA for multiple comparisons was used to determine the *p* value shown on the graph. The data are presented as the means ± SDs. *N* = 3 independent islet isolations and treatments, with between 7 and 25 islets per treatment.

We next evaluated whether MMP2-induced β-cell proliferation and maturation can be maintained over the long term. Over a 35-day period, SC-β-cells were cultured either in basal medium or in medium supplemented with MMP2 and passaged (P) every 7 days (Fig. 6a). To do so, we dissociated SC-β-cell spheroids, followed by endocrine cell quantification via flow cytometry, and replated the cells on low-attachment plates to allow them to reorganize into spheroids. At P6, we detected a marked 172% increase in the number of C-PEP^+^ cells in the presence of MMP2 (Fig. 6b). We also noted a gradual increase in the percentage of C-PEP^+^ cells at each passage: 50% at P1 and 90% at P6 (Fig. 6c). No significant increase in the number of GCG^+^ cells was detected (Fig. 6d). qRT‒PCR confirmed the sustained expression of *INS*, *CHGA, NKX6-1*, and *SUR1* in SC-β-cells cultured with MMP2 (Fig. 6e) and no increase in other islet cell markers, such as *ARX1, HHEX* and *SST* (Supplementary Fig. 6a). Furthermore, short- and long-term SC-β-cell expansion with MMP2 caused increased INS secretion when the cells were exposed to a glucose gradient (Fig. 6f). Upon high glucose stimulation, SC-β-cells subjected to long-term MMP2 treatment secreted more INS than short- and long-term cultured cells did in basal medium (Fig. 6f). After P6, the MMP2-treated SC-β-cells contained more INS than did the untreated cells (Supplementary Fig. 6b). To assess in vivo functionality, we transplanted 1.5 × 10^6^ SC-β-cells, either unexpanded or expanded with MMP2, under the kidney capsules of SCID-Beige mice. After 3 weeks, we probed for human C-peptide in mouse blood serum after fasting or glucose stimulation (Fig. 6g). The serum C-PEP levels were almost twofold greater in the mice with MMP2-treated β-cell implants than in the controls and were similar to those in the mice with human primary islets (~700 IEQ) (Fig. 6h, i). We detected no human C-PEP in the mice injected with saline under the kidney capsule (data not shown). Furthermore, we conducted GTTs on mice transplanted with untreated and MMP2-treated SC-β-cells (Fig. 6j), which revealed that MMP2 treatment lowered blood glucose levels during the GTT. To evaluate the capacity of MMP2-expanded SC-β-cells to maintain euglycemia, we performed a GTT following the STZ-induced loss of endogenous mouse β-cells. In the control group, the mice underwent sham surgery without SC-β-cell transplantation, followed by STZ injection to induce hyperglycemia, as confirmed by fasting blood glucose levels (Supplementary Fig. 6c). The increase in blood glucose during the GTT was attenuated in mice with MMP2-treated SC-β-cells both before and after STZ treatment (Fig. 6j), indicating that the MMP2-treated SC-β-cells effectively preserved euglycemia even after STZ treatment. Concomitantly, with lower glucose spikes during the GTT, we detected higher amounts of human C-peptide in the mice with MMP-treated SC-β-cells (Supplementary Fig. 6d). Finally, we performed nephrectomy to remove the kidney with grafts, resulting in a return to hyperglycemia (Fig. 6k). Thus, we concluded that serial passaging in the presence of MMP2 enhances SC-β-cell function in vivo, which is consistent with our in vitro observations.

We next asked whether MMP2, alone or synergistically with a WNT activator, promotes the proliferation of adult β-cells or other islet cells. Human primary islets were treated with rh MMP2, CHIR99021, or their combination for seven days, but no increase in the proliferation of β-, α- or δ-cells was detected (Fig. 6l, m). Additionally, we assayed the mitogenic effects of MMP2 or CHIR99021 on murine adult whole or dispersed islets and observed enhanced Ins^+^ cell proliferation only after CHIR99021 treatment but not after MMP2 treatment (Supplementary Fig. 6e, f). Together, these data suggest that MMP2 acts as a mitogen specifically for human fetal-like β-cells, implying that the signals regulating the proliferation of newly formed and adult β-cells differ.

### SPOCK2 regulates human β-cell proliferation via the integrin pathway

**Fig. 7:**
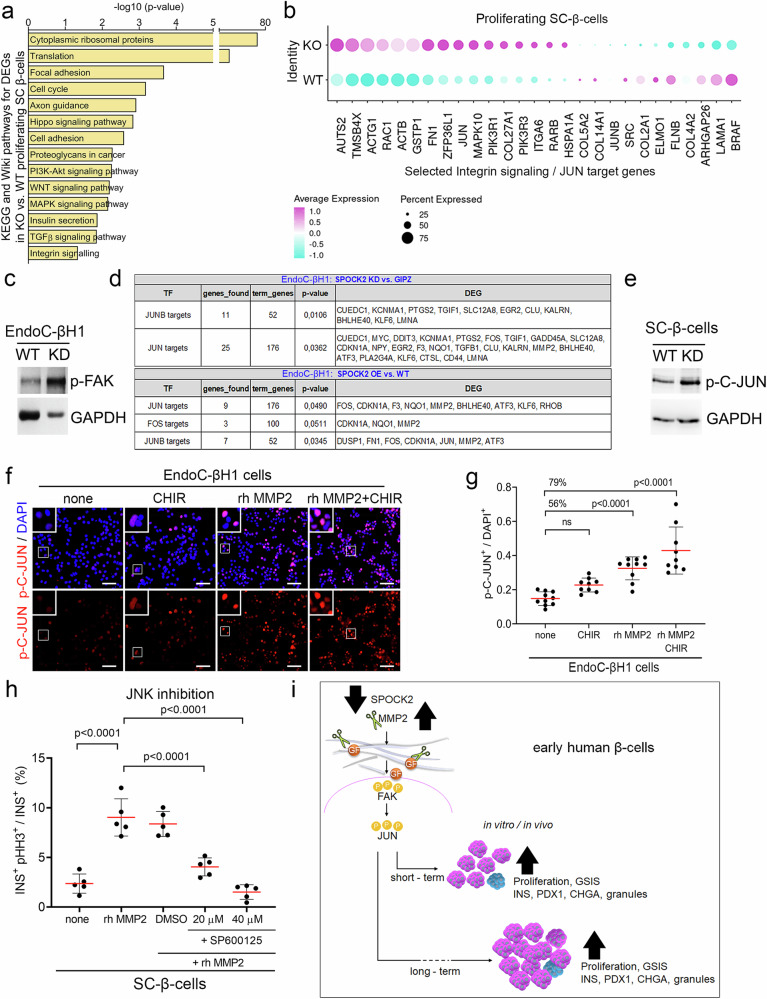
Integrin signaling and early response genes regulate SPOCK2-dependent human β-cell proliferation. **a** Graphical representation of the top-ranking enriched (*p* value ≤ 0.05) KEGG and Wiki pathways among DEGs in proliferating SPOCK2 KO SC-β-cells. **b** Bubble plot showing the relative expression of selected integrin signaling and JUN target genes in proliferating SPOCK2-KO and WT SC-β-cells. The bubble size represents the percentage of expressing cells, and the color intensity reflects average scaled gene expression (pink for upregulation, turquoise for downregulation). **c** Western blot analysis of phospho-FAK protein levels in WT or SPOCK2-KD EndoC-βH1 cells. An antibody against GAPDH was used as a loading control. **d** Differentially expressed AP1 targets identified via RNA-seq analysis of SPOCK2-KD vs. GIPZ and SPOCK2-OE vs. WT EndoC-βH1 cells. **e** Phospho-c-JUN protein levels in WT and SPOCK2-KD SC-β-cells, as determined by western blotting. An antibody against GADPH was used as a loading control. **f** Representative fluorescence microscopy images of EndoC-βH1 cells treated with CHIR, rh MMP2 or rhMMP2 together with CHIR and stained with an antibody against phospho-c-JUN (p-c-JUN red). Examples of p-c-JUN nuclear detection with DAPI (blue) costaining of the nuclei are presented in insets in the top right corners of the merged images. Scale bars = 100 μm. **g** Quantification of phospho-c-JUN^+^ EndoC-βH1 cells treated with CHIR, MMP2 or MMP2 together with CHIR, shown as the ratio of cells expressing the protein to the total number of DAPI^+^ cells. The larger number of EndoC-βH1 cells expressing phospho-c-JUN after MMP2 + CHIR treatment than WT cells expressing this protein are shown as a percentage. One-way ANOVA for multiple comparisons was used to determine the *p* values shown on the graph. The data are presented as the means ± SDs. *N* = 3 biological replicates. **h** Treatment with the JUN/JNK small molecule inhibitor SP600125 diminished MMP2-induced SC-β-cell proliferation. SC-β-cells were treated for 7 days with MMP2 (15 ng/ml), MMP2 + DMSO or MMP2 with 20 μM or 40 μM SP600125, after which the cells were stained for INS and pHH3 and quantitatively analyzed via flow cytometry. The data are presented as the means ± SDs. *N* = 6 biological replicates. One-way ANOVA for multiple comparisons was used to determine the *p* values shown on the graph. **i** Proposed molecular mechanism underlying SPOCK2-dependent β-cell proliferation and function. Reduced *SPOCK2* expression or MMP2 treatment induces human β-cell proliferation via β-integrin-JUN pathway activation. In β-cells, SPOCK2 regulates MMP2, inhibiting its function. When SPOCK2 is knocked down, MMP2 cleaves ECM components, including collagen IV and fibronectin. This releases growth factors and ECM fragments, which act as substrates for the β-integrin receptor. Upon activation, the integrin receptor induces FAK autophosphorylation at Y397, activating downstream effectors, including c-JUN, leading to cell proliferation, the upregulation of β-cell markers (INS, PDX1, CHGA) and an increased number of insulin granules. Consequently, GSIS is improved in the short-term and long-term β-cells both in vitro and in vivo.

We next investigated the molecular mechanism underlying SPOCK2- and MMP2-mediated β-cell proliferation. The top-ranked enriched KEGG and Wiki pathways in KO SC-β-cells included the MAPK, PI3K-Akt, TFG-β, integrin signaling, and focal adhesion pathways (Fig. 7a). As anticipated, we also detected strong enrichment in terms related to positive regulation of the cell cycle and INS secretion (Fig. 7a). In accordance with this observation, analysis of 86 overlapping genes whose expression was increased in KD and downregulated in OE EndoC-βH1 (Supplementary Fig. 7a and Supplementary Table 1) revealed similar KEGG and Wiki pathway terms (Supplementary Fig. 7a, b and Supplementary Table 1). Specifically, we detected altered expression of matrix metallopeptidase 14 (*MMP14*), SRC, collagens, integrin subunit beta 1 *(IGTB1)*, and *JUN*, suggesting alterations in β-integrin signaling (Fig. 7b for KO and Supplementary Fig. 7c–f for KD and OE). MMP2 can bind to integrins and regulate integrin signaling^70^. Based on these findings, we hypothesized that SPOCK2 KD/KO activates the integrin pathway in fetal-like β-cells. We confirmed integrin signaling activation by detecting increased levels of phosphorylated FAK (Y397) in SPOCK2-KD Endo-βH1 cells (Fig. 7c and Supplementary Fig. 7g). We also observed the altered expression of a cohort of integrin effectors, JUN, and targets^71^, including c-JUN and Kruppel-like factor 6 (*KLF6*), in SPOCK2-KO or KD/OE cells (Fig. 7b for KO and 7d for KD or OE). Consistently, we detected increased levels of activated c-JUN protein in SPOCK2-KD SC-β-cells (Fig. 7e and Supplementary Fig. 7h). Immunostaining also revealed increased levels of phosphorylated c-JUN in SPOCK2-KD EndoC-βH1 cells (Supplementary Fig. 7i, j). Treatment with MMP2 alone or in combination with CHIR99021 also increased the number of p-JUN^+^ EndoC-βH1 cells (Fig. 7f, g). Finally, we tested whether ablation of c-JUN phosphorylation by the JNK inhibitor SP600125 would impact SC-β-cell proliferation. The simultaneous treatment of SC-β-cells with MMP2 and SP600125 resulted in a dose-dependent decrease in the percentage of pHH3^+^/INS^+^ cells (Fig. 7h).

Together, these data suggest that the following molecular mechanism regulates fetal-like β-cell proliferation and maturation: When SPOCK2 is abundant in the ECM surrounding β-cells, it inhibits MMP2 expression and activity. In SPOCK2-KD/KO cells, MMP2 cleaves ECM components, including gelatin and collagens. The resulting collagen peptides are substrates for β-integrin receptors^72,73^^,^ and following integrin receptor activation, FAK undergoes autophosphorylation and can then form a complex with SRC, ultimately leading to the phosphorylation of downstream effectors, such as c-JUN, and human β-cell proliferation and/or function^74^. Moreover, both short- and long-term SPOCK2 deficiency or MMP2 treatment led to improved β-cell function (Fig. 7i).

## Discussion

During development, differentiation and expansion occur sequentially. One potential strategy to increase the number of in vitro-derived β-cells is to increase the replication of newly differentiated β-cells. In this context, we identified SPOCK2 as a negative regulator of fetal-like β-cell proliferation in humans. The molecular mechanisms underlying the enhanced replication potential of human fetal β-cells are not yet well understood. Several distinctions exist in the mechanisms regulating the proliferation of embryonic and adult β-cells. For example, adult β-cells strongly express the cell cycle inhibitor p16Ink4a, while the expression of cyclins D1 and D2 decreases with β-cell aging^75,76^. Furthermore, connective tissue growth factor controls β-cell proliferation during development but not in adulthood^77^. Mitogens of adult human β-cells, such as harmine, do not elicit significant replication in fetal β-cells, as represented (albeit with limitations) by the EndoC-βH1 line^34^. We and others^69^ have shown that treatment with the GSK3β inhibitor CHIR99021 induces the replication of up to 15% of EndoC-βH1 cells. In contrast, in adult β-cells, CHIR99021 alone does not induce expansion, and in combination with harmine, it causes only ~3.5% of cells to proliferate^19^.

The differences in proliferative capacity between early and adult β-cells may be attributed, at least in part, to signals from the ECM. Heparan sulfate proteoglycans such as SPARC (another member of the SPARC family to which SPOCK2 belongs^78^) are known to regulate the expression of genes involved in pancreatic β-cell differentiation and GSIS function and to contribute to the maintenance and enhancement of GSIS-responsive function^78,79^.

We investigated which cell types produce SPOCK2 in the developing pancreas. Our findings that the SPOCK2 protein is present in human fetal pancreatic progenitors, β-cells, and δ-cells. Analysis of our scRNA-seq data from the developing murine pancreas revealed the presence of SPOCK2 mRNA in the EP and newly formed β-cells. Further analysis of publicly available scRNA-seq data from the fetal pancreas at week 16–20 confirmed the presence of SPOCK2 mRNA in EPs, β-cells and subclusters of acinar cells. SPOCK2 is also expressed in endothelial cells at week 8–12^80^.

RNA-seq analyses of human β-cells with dysregulated SPOCK2 levels revealed altered *MMP2* expression. Interestingly, MMP2 has been identified as a marker of newly formed immature β-cells, as MMP2 protein is present in rat β-cells at E20.5 and P2 (postnatal Day 2) but is no longer detectable after P7^81^. In mice treated with a low dose of STZ, MMP2 expression is upregulated in regenerating β-cells^82^. Furthermore, MMP2 activity modulates proper islet morphogenesis in rat pancreatic rudiments cultured in a 3D collagen matrix, and its regulation is tightly controlled by TGF-β signaling. In the presence of exogenous TGF-β, both islet morphogenesis and MMP2 activation are accelerated. When MMP2 activity is inhibited, endocrine cell differentiation proceeds normally; however, islet morphogenesis is perturbed^83^. Interestingly, our RNA-seq data revealed alterations in TGF-β signaling, suggesting that SPOCK2 KD might also impact this pathway in parallel with integrin signaling. SPOCK2 and MMP2 can interact^84^, SPOCK2 is predicted to have an MMP-binding domain^63,85^, and MMP2 contains a binding site for the SPARC family^63^. This interaction may limit MMP2 enzymatic activities either directly or through other regulators. MMP2 activity is positively regulated by MMP14 and negatively regulated by TIMP1 or TIMP2^66,86,87^. In SPOCK2-KD EndoC-βH1 cells, *TIMP1* is downregulated, and *MMP14* is upregulated. Moreover, MMP2 activity requires the presence of integrin receptors in proximity to the MMP2-MMP14 complex or even their direct binding^86,88^. We found that several collagens, including *COL4A5* and *COL4A2*, as well as fibronectin, were differentially expressed between SPOCK2 KD/KO and OE β-cells. Therefore, we propose a molecular mechanism controlling early β-cell proliferation in humans, in which SPOCK2 limits MMP2 activity either directly or indirectly. Upon SPOCK2 silencing, MMP2 activity is no longer restricted, and MMP2 can cleave ECM components, including collagens and fibronectin, leading to the activation of integrin signaling pathways. In SPOCK2-KD human β-cells, FAK, an integrin receptor effector, is activated along with c-JUN, a well-known regulator of cell cycle progression^89^. Furthermore, several β-integrin pathway components, including *ITGB1*, *PAX4*, *PAX5*, and *TLN2*, are differentially expressed following the modulation of SPOCK2 levels in β-cells.

It is probable that SPOCK2 dysregulation can influence the activity of other growth factors or signaling pathways due to the presence of multiple growth factors in the ECM, where they are sequestered and locally concentrated to act on β-cells or other cell types^90^. Indeed, our analysis of RNA-seq datasets revealed the activation of other signaling pathways, such as the PI3K-Akt, MAPK or TGFβ pathways, in SPOCK2-deficient early β-cells. However, the findings that SPOCK2 deficiency results in a 46% increase in the number of proliferating β-cells and that MMP2 treatment results in a 33% increase in replication suggest that MMP2 plays an important role in mediating SPOCK2-dependent β-cell expansion.

The adult β-cell population is heterogeneous, and some of these subpopulations display varying proliferative capacities. In humans, LIFR^+^ β-cells^23^ and β-cells lacking Flattop, an effector of Wnt/planar cell polarity signaling^91^, have been identified as proliferation-competent subpopulations. Another distinct subpopulation, comprising so-called “virgin” β-cells, characterized by the absence of Ucn3, has been described in mice as a presumptive β-cell neogenesis niche^92^. It has been proposed that Ucn3^-^ cells represent an intermediate stage in transdifferentiation between α and β-cells and have a greater proliferation capacity^92^. However, it remains to be determined whether similar proliferation-prone subpopulations exist within the newborn human β-cell population.

Intriguingly, several studies have suggested that a higher proliferative capacity of adult β-cells may be correlated with the physiological immature phenotype^93–95^, including a lack of glucose responsiveness. We consistently found that SPOCK2 deficiency in SC-β-cells and EndoC-βH1 increased their GSIS and led to a regulated insulin secretion profile equivalent to that of primary islets. These data might indicate that human fetal-like β-cells can be induced to proliferate while maintaining optimal physiological capacity or that SPOCK2 separately regulates both the proliferation and function of β-cells. Given the expression of SPOCK2 in EPs and potentially even BPs, coupled with the improved function of SPOCK2-deficient β-cells, further investigation is warranted to elucidate the potential role of SPOCK2 in β-cell development, maturation and functional regulation. As ~20% of SPOCK2-KO SC β-cells actively proliferate, single-cell assays are needed to assess the functionality of the proliferating β-cells or whether the improvement in GSIS is due to the ability of SPOCK2 to affect different β-cell subpopulations. It would also be interesting to know whether MMP2 is necessary to maintain SC-β-cell function or, similarly, if the effect of MMP2 on proliferation is reversible.

In conclusion, the transcriptomic and physiological profiles and proliferation capacity of adult and newborn β-cells differ. Newly formed β-cells, while already committed, have greater replication potential and are immature. Such transient immature states occur not only in developing and neonatal β-cells in humans^25,26^ but also in newly differentiated SC-β-cells in vitro^29,30^ during pathological processes or regeneration. The mechanisms driving the proliferation of early β-cells could be reactivated in adult cells to increase the functional β-cell mass, especially during periods of high metabolic demand, such as pregnancy, weight gain, decreased insulin sensitivity or experimentally STZ-induced hyperglycemia. Enhanced regenerative capacity is also observed in newly formed cell types within other tissues and organs. For example, the neonatal heart regenerates efficiently following injury, but this regenerative capacity is lost within seven days in mice or one month in humans^96,97^. Similarly, wound healing is more efficient and scarless in developing embryos than in adult animals^98^. An improved understanding of the signals controlling the regeneration of newly formed or neonatal cells could pave the way for the robust derivation of functional human cells in vitro.

## Supplementary information

Supplementary Table 1

Supplementary information

Supplementary Video
