# Supplementary material for: SPOCK2 controls the proliferation and function of immature pancreatic β-cells through MMP2
Source: Exp Mol Med. 2025 Jan 1;57(1):131–50. doi: 10.1038/s12276-024-01380-2 (PMC11799530; doi:10.1038/s12276-024-01380-2)
Supplement: Supplementary file 2 — Supplementary information [file 12276_2024_1380_MOESM2_ESM.pdf]

Supplementary Fig. 1

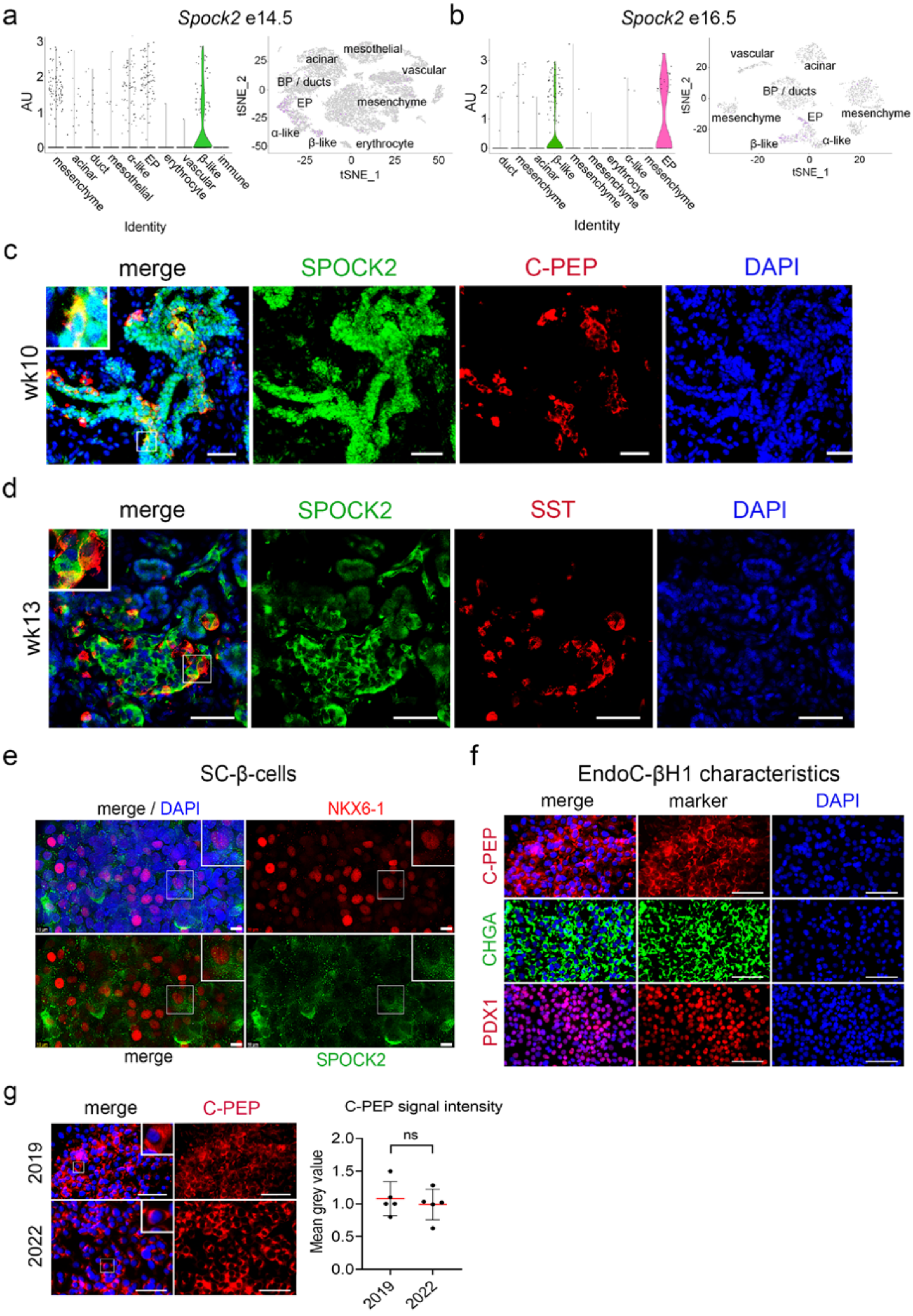

**Supplementary Figure 1.**

**SPOCK2 is expressed in the human fetal pancreas and in the human fetal β-cell line EndoC-βH1.**

**a. Left -** Violin plot represents the expression of mouse *Spock2* in different cell clusters from e14.5 pancreas. **Right -** tSNE plot shows *Spock2* transcript levels (dark violet – high levels; light violet – low levels; gray – no expression) in e14.5 pancreas. Cell clusters were annotated for the expression of known markers.

**b. Left -** Violin plot represents the expression of mouse *Spock2* in different cell clusters from e16.5 pancreas. **Right -** tSNE plot shows *Spock2* transcript levels (dark violet – high levels; light violet – low levels; gray – no expression) in e16.5 pancreas. Cell clusters were annotated for the expression of known markers.

**c.** Human fetal (Wk10) pancreas stained with antibodies against SPOCK2 (green) and C-PEPTIDE (C-PEP, red) labeling early β-cells. DAPI (blue) was used to stain the nuclei. Scale bars = 100 µm. Inset – co-localization is shown in yellow, marking the area bordered by the rectangle (merge panel).

**d.** Human pancreas (Wk13) stained with antibodies against SPOCK2 (green) and SOMATOSTATIN (SST, red) labeling early δ-cells. DAPI (blue) was used to stain the nuclei. Scale bars = 100 µm. Inset – co-localization is shown in yellow, marking the area bordered by the rectangle (merge panel).

**e.** Representative fluorescence microscopy images of hPSC-derived β-cells (SC-β-cells) stained with antibodies against SPOCK2 (green) and NKX6-1 (red). DAPI (blue) marks the nuclei. Scale bars = 10 µm. An example of protein co-expression is presented in the inset in the top left corner of the merged image.

**f.** Representative fluorescence microscopy images of EndoC-βH1 cells stained with antibodies against C-PEPTIDE (C-PEP, red) – top panel, CHROMOGRANIN A (CHGA, green) – middle panel, and PDX1 (red) – bottom panel. DAPI (blue) was used to stain the nuclei. Scale bars = 100 µm. DAPI (blue) was used to stain the nuclei.

**g. Left -** Representative fluorescence microscopy images of EndoC-βH1 cells stained with an antibody against C-PEP (red) of early (year 2019) and late (year 2022) passages. DAPI (blue) was used to stain the nuclei. Scale bars = 100 µm. Inset- higher magnification of the square-bordered area (merge panel). **Right -** Quantification of fluorescence signals with mean gray value determination, showing no difference in C-PEP protein levels between EndoC-βH1 cells of early and late passages. t-Student test was used to determine the p-values shown on the graph. Data are presented as means ± SD. N = 5 biological replicates.

**Supplementary Fig. 2**

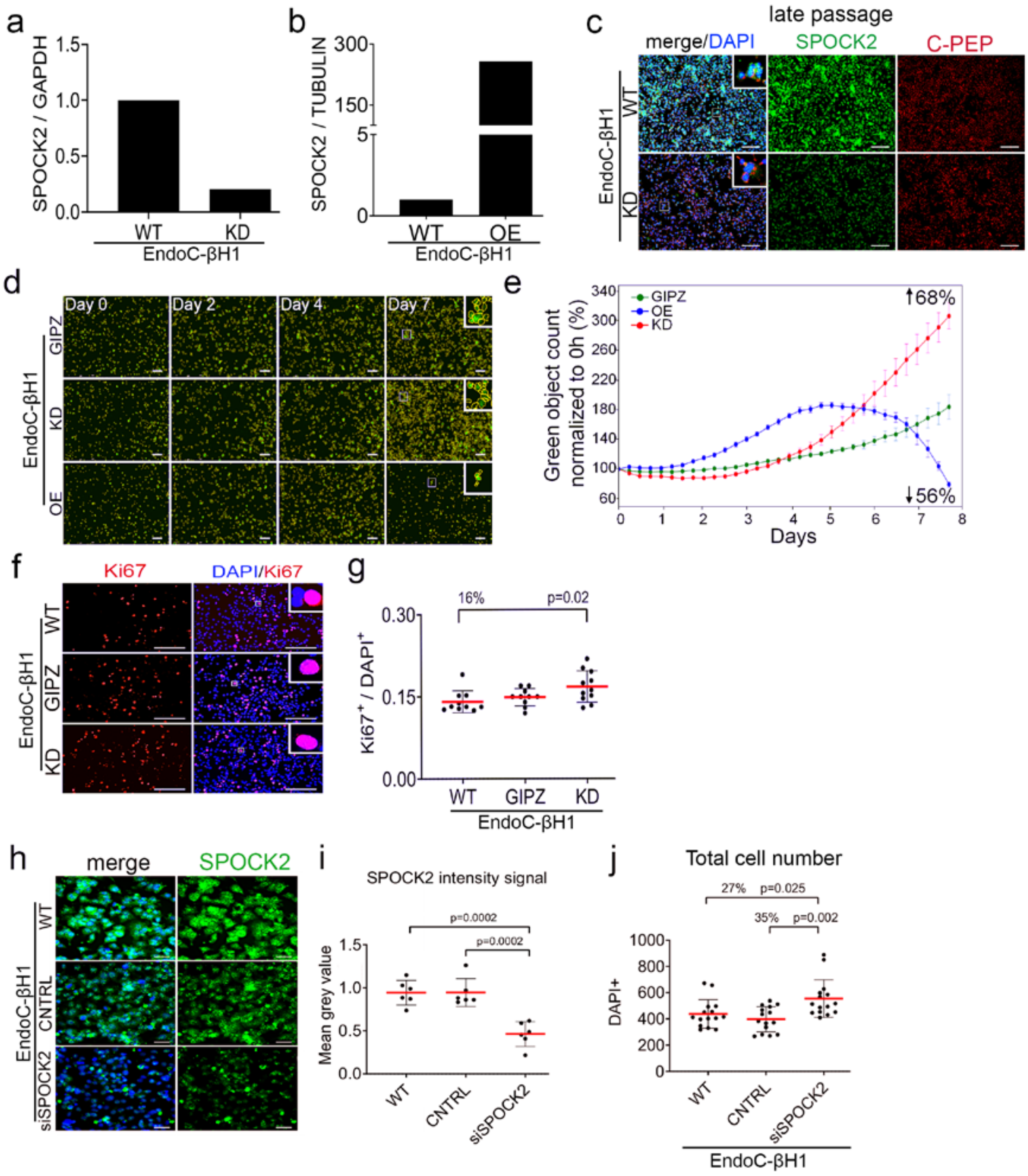


**Supplementary Figure 2.**

**Bidirectional modulation of SPOCK2 expression controls early β-cell number.**

**a.** Quantification of western blots from Fig. 2D showing the lower levels of SPOCK2 protein in EndoC-βH1 SPOCK2 KD cells than in WT cells. Abundance of GAPDH was used for signal normalization.

**b.** Quantification of western blots from Fig. 2G showing the higher SPOCK2 protein levels in EndoC-βH1 SPOCK2 OE cells compared to WT cells. Abundance of β-tubulin was used for signal normalization.

**c.** Representative fluorescence microscopy images of EndoC-βH1 SPOCK2 KD and WT cells from the late passage stained with antibodies against SPOCK2 (green) and C-PEP (red), labeling β-cells and showing the lower level of SPOCK2 protein in SPOCK2 KD cells than in WT cells. DAPI (blue) marks the nuclei. Scale bars = 100 µm.

**d.** Representative images from cell cultures on days 0, 2, 4, and 7, showing the differences in the number of $GFP^+$ cells (marked by a yellow outline) between EndoC-βH1 GIPZ, SPOCK2 KD, and SPOCK2 OE cells. Scale bars = 100 μm.

**e.** Quantification of green object counts over an eight-day time course normalized against the 0 h time point, showing higher number of SPOCK2 KD (red) and lower number of SPOCK2 OE (blue) cells relative to GIPZ controls (dark green). *N* = 3 biological replications.

**f.** Representative fluorescence microscopy images of EndoC-βH1 WT (top), GIPZ control (middle), and SPOCK2 KD (bottom) cells stained with an antibody against the proliferation marker Ki67 (red). Examples of Ki67 nuclear detection after co-staining with DAPI (blue), a nuclear marker, are presented as insets in the top right corner of the merged images. Scale bars = 100 μm.

**g.** Quantification of $Ki67^+$ cells among EndoC-βH1 WT, GIPZ control, and SPOCK2 KD cells, presented graphically as the ratio of $Ki67^+$ cells to the total number of live cells ($DAPI^+$). The greater number of SPOCK2 KD cells expressing pHH3 than that of WT and GIPZ controls is given as a % of change. Each dot on the graph represents a single image. A one-way ANOVA for multiple comparisons was used to determine the *p*-values shown on the graph. Data are presented as means ± SD. *N* = 10-11 biological replicates.

**h.** Representative fluorescence microscopy images of EndoC-βH1 WT (top), siRNA control (middle), and siRNA SPOCK2 KD cells (bottom) immunostained with antibody against SPOCK2 (green). The lower levels of SPOCK2 protein in siRNA SPOCK2 KD cells as compared to WT and siRNA control cells are seen. DAPI (blue) was used to stain the nuclei. Scale bars = 100 μm.

**i.** Quantification of fluorescence signals from Supplementary Fig. 2H with mean gray value determination, showing the significantly lower SPOCK2 protein levels in EndoC-βH1 siRNA SPOCK2 KD cells relative to WT and siRNA controls. A one-way ANOVA for multiple comparisons was used to determine the *p*-values shown on the graph. Data are presented as means ± SD. N = 6 biological replicates.

**j.** Quantification of the total number of cells ($DAPI^+$) in EndoC-βH1 WT, siRNA control, and siRNA SPOCK2 KD cells. The increase in the number of siRNA SPOCK2 KD cells, compared to WT and siRNA controls is given as a % of change. A one-way ANOVA for multiple comparisons was used to determine the *p*-values shown on the graph. Data are presented as means ± SD. *N* = 3 biological replicates and 5 images were quantified per each replicate.

**Supplementary Fig. 3**

**Supplementary Figure 3.**

**SPOCK2 regulates the early β-cell proliferation.**

**a.** FACS analysis of hPSC (H1)-derived β-cells stained with the antibodies against β-cell surface marker, CD49a conjugated to GFP-A (*x*-axis) and α-cell surface marker-TM4SF4 conjugated to APC-A (y-axis) showing the % of β-cells (gate P4) and α-cells (gate P5).

**b.** Quantification of double-positive pHH3$^+$/CD49a$^+$ and TM4SF4$^-$ cells after treatment with rh SPOCK2 protein for seven days. The hPSCs were differentiated to β-cells and β-cells were enriched based on CD49a cell surface protein expression, treated with different concentrations of rh SPOCK2 followed by staining against pHH3. The data were normalized to the untreated control. A one-way ANOVA for multiple comparisons was used to determine the *p*-values. Data are presented as means ± SD. $N$ = 5 biological replicates.

**c.** Representative fluorescence microscopy images of EndoC-βH1 WT cells treated for seven days with recombinant human SPOCK2 (rh SPOCK2; 2 µg/mL) and stained with an antibody against pHH3 (red). Untreated WT cells were used as a control. DAPI (blue) marks the nuclei. Scale bars = 100 µm.

**d.** Quantification of pHH3$^+$ EndoC-βH1 WT cells treated for seven days with various concentrations of rh SPOCK2 (0.5 µg/mL, 1 µg/mL, or 2 µg/mL), presented as the ratio (in %, one-way ANOVA statistics) of pHH3$^+$ cells to the total number of live cells (DAPI$^+$). Data are presented as means ± SD. $N$ = 3 biological replicates and 8-12 images per replicate were quantified.

**e.** Representative fluorescence microscopy images of SC-β-cells transduced with the *SPOCK2* shRNA virus containing GFP (green). BF-bright field. Scale bars = 100 µm.

**f.** Representative fluorescence microscopy images of WT and SPOCK2 KO hPSC-derived SC-β-cells stained with antibodies against SPOCK2 (green) and NKX6-1 (grey). DAPI (blue) marks the nuclei. Scale bars = 50 µm.

**Supplementary Fig. 4**

**Supplementary Figure 4.**

**SPOCK2 improves functionality of early β-cell.**

**a.** Heatmap of β-cell function-related genes differentially expressed between EndoC-βH1 SPOCK2 KD and GIPZ control cells in the RNA-seq experiment indicating the improved β-cell physiology in EndoC-βH1 SPOCK2 KD cells.

**b. Left** - Representative fluorescence microscopy images of EndoC-βH1 WT and SPOCK2 KD cells stained with antibodies against C-PEPTIDE (red). Scale bars = 100 μm. DAPI (blue) marks the nuclei. **Right** - Quantification of fluorescence signals from C-PEP staining with mean gray value determinations, showing the significantly higher protein levels in EndoC-βH1 SPOCK2 KD cells relative to WT. Data are presented as means ± SD. t-Student test was used to determine the *p*-values shown on the graph. *N* = 12 biological replicates.

**c. Left** - Representative fluorescence microscopy images of EndoC-βH1 WT and SPOCK2 KD cells stained with antibodies against CHGA (red). DAPI (blue) marks the nuclei. Scale bars = 100 μm. **Right** - Quantification of fluorescence signals from CHGA staining with mean gray value determinations, showing the significantly lower both protein levels in EndoC-βH1 SPOCK2 KD cells relative to WT. t-Student test was used to determine the *p*-values shown on the graph. Data are presented as means ± SD. *N* = 14 biological replicates.

**d.** Insulin secretion in EndoC-βH1 SPOCK2 KD and WT cells challenged with low glucose (2.8 mM), or high glucose (16.7 mM) normalized to the total cell number. A one-way ANOVA for multiple comparisons was used to determine the p-values shown on the graph. Data are presented as means ± SD. *N* = 6 biological replicates.

**e.** Insulin stimulation index, calculated as a ratio of insulin secreted after 16.7 mM to basal (2.8 mM) glucose treatment, for EndoC-βH1 WT and SPOCK2 KD cells, showing the higher stimulation index for SPOCK2 KD EndoC-βH1 cells. A one-way ANOVA for multiple comparisons was used to determine the *p*-values shown on the graph. The data are presented as means ± SD. *N* = 6 biological replicates.

Supplementary Fig. 5

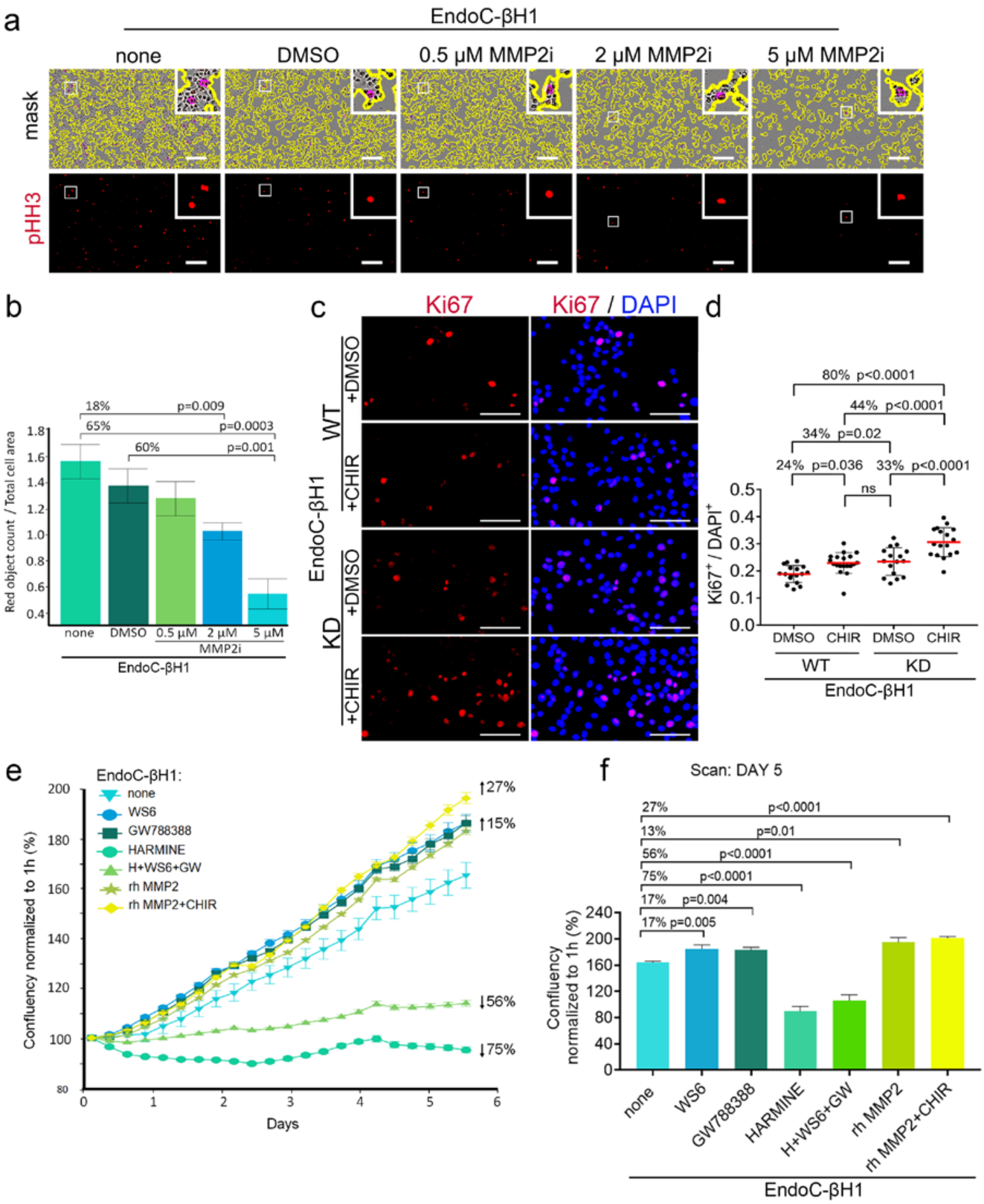


**Supplementary Figure 5.**

**SPOCK2-mediated MMP2 activation increases EndoC-βH1 and SC-β-cell proliferation.**

**a.** Representative bright-field images with cells marked by a yellow mask (top panel) and fluorescent microscope images of EndoC-βH1 WT cells stained with an antibody against the proliferation marker pHH3 (bottom panel, red). EndoC-βH1 WT cells treated for five days with different MMP2i concentrations showed lower confluence and numbers of pHH3$^{+}$ cells compared to WT and DMSO-treated cells. Scale bars = 100 µm.

**b**. Quantification of $pHH3^+$ cells among EndoC-βH1 WT cells treated for five days with different concentrations of MMP2i, shown as the ratio between $pHH3^+$ cells (red object count) and total cell area. The decrease in the number of EndoC-βH1 WT cells expressing pHH3 after MMP2i treatments compared to untreated controls is shown as a %. One-way ANOVA for multiple comparisons was applied to determine *p*-values, shown on the graph. Data are presented as mean ± SD. *N* = 3 biological replications.

**c.** Representative fluorescence microscopy images of EndoC-βH1 WT and SPOCK2 KD cells after pretreatment for 48 h with 10 µM CHIR992021 (CHIR) or DMSO (control) and staining with an antibody against Ki67. DAPI (blue) marks the nuclei. Scale bars = 50 µm.

**d.** Quantification of $Ki67^+$ cells among EndoC-βH1 WT and SPOCK2 KD cells, shown graphically as the ratio of $Ki67^+$ cells to the total number of live cells ($DAPI^+$). The larger number of EndoC-βH1 WT and SPOCK2 KD cells expressing Ki67 after treatment with 10 µM CHIR than for DMSO-treated control cells shown as a %. A one-way ANOVA for multiple comparisons was used to determine the *p*-values shown on the graph. The data are presented as means ± SD. *N* = 4 biological replications and 6-8 images per replicate were quantified.

**e.** Comparison of MMP2 induced human β-cell proliferation to other known β-cell mitogens. EndoC-βH1 WT cells were either none-treated, treated with different human β-cell mitogens, including harmine, WS6 and GW78838, MMP2 or MMP2 and CHIR99021 and total confluence was live-monitored over a five-day long culture.

**f**. Confluence quantification from images taken on day 5 and normalized to the 1 h time point in the cell culture of EndoC-βH1 WT either none-treated, treated with different human β-cell mitogens, including harmine, WS6 and GW78838, MMP2 or MMP2 and CHIR99021. A one-way ANOVA for multiple comparisons was used to determine the *p*-values shown on the graph. Data are presented as means ± SD. *N* = 3 biological replicates.

**Supplementary Fig. 6**

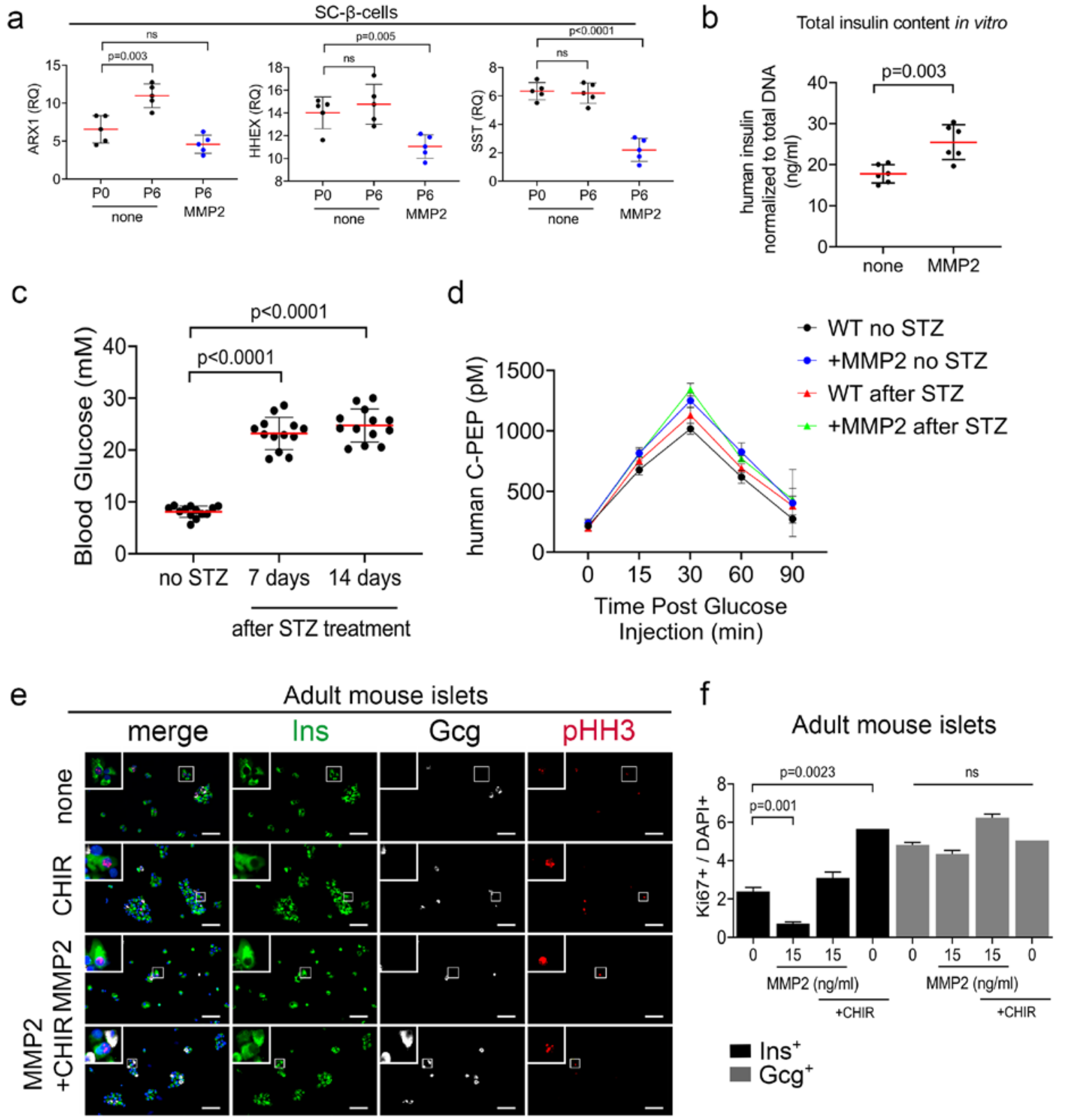


**Supplementary Figure 6.**

**Long-term serial expansion of SC-β-cells with MMP2 protein results in improved insulin secretion *in vitro* and *in vivo*.**

**a.** qRT-PCR analysis of mRNA expression of pancreatic α- and δ-cell markers.

**b.** Normalized total insulin content of MMP2-treated and untreated SC-β-cells.

**c.** Blood glucose levels in control SCID-Beige mice before and after STZ-induced diabetes. Mice were fasted for 16 h and blood glucose was subsequently measured. Data are presented as means ± SD. N = 13 mice. p-value was determined using t-test.

**d.** ELISA measurements of human C-PEP levels in mice before and after STZ-induced diabetes, transplanted with rh MMP2 treated (+MMP2) or non-treated (WT) SC-β-cells. Mice were fasted for 16 h and hC-PEP was subsequently measured before (0 min) and 15, 30, 60 and 90 min after a glucose injection. Data are presented as means ± SD. N = 4 mice.

**e.** Mouse adult islet cell proliferation after MMP2 and Wnt activation. Mouse adult islets were treated with MMP2 and CHIR99021 alone or in combination, over seven days and then stained for Ins, Gcg and pHH3. Representative confocal images of staining against Ins (green), Gcg (white) and pHH3 (red) are shown. DAPI marks nuclei in blue. Scale bars = 100 µm.

**f.** The quantification of proliferating (Ki67+) Ins+ β-cells and Gcg+ α-cells after 7-day long treatment with 15 ng/mL MMP2 alone or in combination with 10 µM CHIR99021 compared to untreated control. N = 3 independent islet isolations and treatments, with 8-10 mice per replicate. A one-way ANOVA for multiple comparisons was used to determine the p-value shown on the graph. Data are presented as means ± SD. N = 3 independent islet isolations and treatments, with 8-10 mice per replicate.

**Supplementary Fig. 7**

**Supplementary Figure 7.**

**Insights into the mechanism of SPOCK2-mediated human β-cell proliferation.**

**a. Left** panel - Venn diagram of DEGs in human β-cells with SPOCK2 OE or KD; genes upregulated in EndoC-βH1 SPOCK2 KD cells are shown in violet and downregulated in SPOCK2 OE cells in green. The intersection (shared DEGs) is in the light orange. **Right** panel - Graphical representation of the top-ranking enriched *p*-value ≤ 0.05) biological processes and pathways in Wiki and KEGG analyses, respectively, of the shared genes: upregulated in EndoC-βH1 SPOCK2 KD cells and downregulated in SPOCK2 OE cells.

**b.** Graphical representation of the top-ranking enriched p-value ≤ 0.05) KEGG and Wiki pathways among DEGs in EndoC-βH1 SPOCK2 KD vs. GIPZ control cells and EndoC-βH1 SPOCK2 OE vs. WT cells. The number of genes included in each term is reflected in the intensity grade of the violet color and given by each bar.

**c.** Heatmap of integrin signaling-related genes differentially expressed between EndoC-βH1 SPOCK2 OE vs. WT cells in the RNA-seq experiment indicating the decreased expression of integrin signaling associated genes in EndoC-βH1 SPOCK2 OE cells.

**d.** Heatmap of integrin signaling-related genes differentially expressed between EndoC-βH1 SPOCK2 KD vs. GIPZ control cells in the RNA-seq experiment indicating the increased expression of integrin signaling associated genes in EndoC-βH1 SPOCK2 KD cells.

**e.** Enrichment plot from the gene set enrichment analysis (GSEA) of genes differentially expressed between EndoC-βH1 SPOCK2 OE and GIPZ control cells in the RNA-seq experiment compared to the integrin signaling gene set. We performed 1000 permutations in the GSEA analysis. Gene sets with a false discovery rate (FDR) ≤ 0.02 are shown.

**f.** Enrichment plot from the gene set enrichment analysis (GSEA) of genes differentially expressed between EndoC-βH1 SPOCK2 KD and GIPZ control cells in the RNA-seq experiment compared to the integrin signaling gene set. We performed 1000 permutations in the GSEA analysis. Gene sets with a false discovery rate (FDR) ≤ 0.24 are shown**.**

**g**. Quantification of the western blot from Fig. 7C showing the higher levels of phospho-FAK protein in EndoC-βH1cells with SPOCK2 KD than in WT cells.

**h.** Quantification of the western blot from Fig. 7E showing the higher levels of phospho-c-JUN protein in SC-β-cells with SPOCK2 KD than in WT cells.

**i.** Representative fluorescence microscopy images of EndoC-βH1 WT and SPOCK2 KD cells stained with an antibody against c-JUN (red) or phospho-c-JUN (p-c-JUN red). Examples of c-JUN or p-c-JUN nuclear detection with DAPI (blue) co-staining of the nuclei, are presented in insets in the top right corners of the merged images. Scale bars = 100 µm.

**j.** Quantification of c-JUN$^+$ or phospho-c-JUN$^+$ cells among EndoC-βH1 WT and SPOCK2 KD cells, shown as the ratio of cells expressing the protein of interest to the total number of DAPI$^+$ cell. The larger number of EndoC-βH1 SPOCK2 KD cells expressing phospho-c-JUN than of WT cells expressing this protein is shown as a %. A one-way ANOVA for multiple comparisons was used to determine the *p*-values shown on the graph. Data are presented as means ± SD. *N* = 5 biological replicates for WT and 4 for KD.
